# Supplementary material for: Plasma Galectin-3 predicts deleterious vascular dysfunction affecting post-myocardial infarction patients: An explanatory study
Source: PLoS One. 2020 May 11;15(5):e0232572. doi: 10.1371/journal.pone.0232572 (PMC7213735; doi:10.1371/journal.pone.0232572)
Supplement: S1 Protocol — (DOC) [file pone.0232572.s003.doc]

**Protocole de Recherche Clinique**

Avis favorable du CPP Est III le

Autorisation de l’AFSSAPS le

Version **du 01/12/2008**

**ETUDE MULTIPARAMETRIQUE DU REMODELAGE CARDIAQUE APRES INFRACTUS MYOCARDIQUE REVASCULARISE EN PHASE AIGUE : RELATION AVEC LES CONCENTRATIONS SERIQUES EN ALDOSTERONE**

**⮚ Promoteur** :

Centre Hospitalier Universitaire de Nancy

Avenue du Maréchal de Lattre de Tassigny

54035 NANCY Cedex

**⮚ Responsable de la recherche agissant pour le compte du promoteur / et autorisé à signer le protocole et ses modifications éventuelles au nom du promoteur :**

Direction de la Recherche et de l’Innovation

Ph. BOULANGE – Directeur de la Recherche et de l’Innovation

Hôpital Saint Julien – Rue Foller

54035 NANCY Cedex

**⮚ Investigateur principal coordonnateur**

Michael ANGIOI

Service de Cardiologie

Hôpital de Brabois

Téléphone : 03 83 15 32 40

Télécopie : 03 83 15 42 13

m.angioi@chu-nancy.fr

**SOMMAIRE**

[1 RESUME DE LA RECHERCHE 4](#__RefHeading___Toc215909956)

[2 Liste des abreviations 5](#__RefHeading___Toc215909957)

[3 Justification scientifique et description générale de la recherche 6](#__RefHeading___Toc215909958)

[3.1 Aldostérone, infarctus myocardique et insuffisance cardiaque 7](#__RefHeading___Toc215909959)

[3.2 L’IRM pour quantifier les séquelles d’infarctus, le remodelage cardiaque et le fonctionnement vasculaire 9](#__RefHeading___Toc215909960)

[3.3 Biomarqueurs plasmatiques ET ACTIVITE de fibrose SYSTEMIQUE 12](#__RefHeading___Toc215909961)

[4 Objectifs de la recherche 14](#__RefHeading___Toc215909962)

[4.1 Objectif principal 14](#__RefHeading___Toc215909963)

[4.2 Objectif secondaire 14](#__RefHeading___Toc215909964)

[4.3 Critère principal de jugement 14](#__RefHeading___Toc215909965)

[4.4 Critères secondaires de jugement 14](#__RefHeading___Toc215909966)

[5 Conception de la recherche 15](#__RefHeading___Toc215909967)

[5.1 Déroulement de l’étude 15](#__RefHeading___Toc215909968)

[5.2 Description des examens de l’étude 16](#__RefHeading___Toc215909969)

[5.2.1 IRM Cardio-vasculaire 16](#__RefHeading___Toc215909970)

[5.2.2 Biomarqueurs 18](#__RefHeading___Toc215909971)

[5.3 Calendrier des visites 23](#__RefHeading___Toc215909972)

[5.4 Données recueillies 24](#__RefHeading___Toc215909973)

[5.5 Mesures prises pour éviter les biais 24](#__RefHeading___Toc215909974)

[5.6 Durée de l’étude 25](#__RefHeading___Toc215909975)

[5.7 Gestion des arrêts prématurés et des exclusions en cours d’étude 25](#__RefHeading___Toc215909976)

[5.7.1 Gestion des arrêts prématurés 25](#__RefHeading___Toc215909977)

[5.7.2 Exclusions en cours d’étude 25](#__RefHeading___Toc215909978)

[5.7.3 Arrêt de l’étude 25](#__RefHeading___Toc215909979)

[5.8 Participation simultanée à une autre recherche 25](#__RefHeading___Toc215909980)

[6 Sélection et exclusion des personnes de la recherche : 26](#__RefHeading___Toc215909981)

[6.1 Critères d’inclusion 26](#__RefHeading___Toc215909982)

[6.2 Critères de non inclusion 26](#__RefHeading___Toc215909983)

[7 Traitement administré aux personnes qui se prêtent à la recherche 26](#__RefHeading___Toc215909984)

[8 Evaluation de l’efficacité 27](#__RefHeading___Toc215909985)

[9 Evaluation de la sécurité 27](#__RefHeading___Toc215909986)

[9.1 Description des paramètres d’évaluation de la sécurité 27](#__RefHeading___Toc215909987)

[9.2 Méthodes et calendrier prévus pour mesurer, recueillir et analyser les paramètres d’évaluation de la sécurité 27](#__RefHeading___Toc215909988)

[9.3 Procédures mises en place en vue de l’enregistrement et de la notification des évènements indésirables 27](#__RefHeading___Toc215909989)

[9.3.1 Définitions 27](#__RefHeading___Toc215909990)

[9.3.2 Document de référence permettant de définir le caractère attendu d’un EIG 27](#__RefHeading___Toc215909991)

[9.3.3 Liste des effets indésirables attendus 27](#__RefHeading___Toc215909992)

[9.3.4 Transmission des EIG et faits nouveaux 28](#__RefHeading___Toc215909993)

[9.3.5 Transmission des effets indésirables non graves 28](#__RefHeading___Toc215909994)

[9.4 Modalités et durée de suivi des personnes suite à la survenue d’évènements indésirables 28](#__RefHeading___Toc215909995)

[9.5 Comité de surveillance indépendant 28](#__RefHeading___Toc215909996)

[9.6 Rapports de sécurité 28](#__RefHeading___Toc215909997)

[10 Statistiques 29](#__RefHeading___Toc215909998)

[10.1 Considérations générales 29](#__RefHeading___Toc215909999)

[10.1.1 Justificatif de l’effectif 30](#__RefHeading___Toc215910000)

[10.1.2 Variables quantitatives continues 30](#__RefHeading___Toc215910001)

[10.1.3 Variables qualitatives discontinues 30](#__RefHeading___Toc215910002)

[10.2 Analyses réalisées pour répondre à l’objectif principal 31](#__RefHeading___Toc215910003)

[10.3 Analyses réalisées pour répondre aux objectifs secondaires 32](#__RefHeading___Toc215910004)

[11 Droit d'accès aux données et documents source. 32](#__RefHeading___Toc215910005)

[12 Contrôle et assurance de la qualité. 33](#__RefHeading___Toc215910006)

[13 Considérations éthiques 33](#__RefHeading___Toc215910007)

[13.1 FICHIER NATIONAL 33](#__RefHeading___Toc215910008)

[13.2 CPP/AFSSAPS 34](#__RefHeading___Toc215910009)

[13.3 AMENDEMENT (= modification substantielle) 34](#__RefHeading___Toc215910010)

[13.4 CONSENTEMENT 34](#__RefHeading___Toc215910011)

[13.5 Assurance 34](#__RefHeading___Toc215910012)

[13.6 Résultats globaux 35](#__RefHeading___Toc215910013)

[14 Traitement des données et conservation des documents et des données relatives à la recherche 35](#__RefHeading___Toc215910014)

[14.1 Protection des données 35](#__RefHeading___Toc215910015)

[14.2 Archivage des documents 35](#__RefHeading___Toc215910016)

[15 Financement 35](#__RefHeading___Toc215910017)

[16 Règles relatives à la publication. 35](#__RefHeading___Toc215910018)

[17 Liste des annexes 36](#__RefHeading___Toc215910019)

[17.1 Annexe 1 liste des investigateurs 37](#__RefHeading___Toc215910020)

[17.2 Annexe 2 RCP DOTAREM ^TM^ 38](#__RefHeading___Toc215910021)

[17.3 Annexe 3 lettre d’INFORMATION ET DE CONSENTEMENT 43](#__RefHeading___Toc215910022)

[17.4 Annexe 4 Fiche d’eig 50](#__RefHeading___Toc215910023)

# RESUME DE LA RECHERCHE

Après infarctus myocardique, l’évolution vers l’insuffisance cardiaque est généralement liée à une aggravation progressive de la dysfonction cardiaque, le remodelage délétère. Celui-ci dépend avant tout de l’étendue des séquelles d’infarctus, mais aussi d’autres paramètres tels que l’âge et l’hypertension artérielle. L’aldostéronémie mesurée dès les premières heures de l’infarctus est associée à un mauvais pronostic à long terme. L’aldostérone interagit à la fois sur les mécanismes de cicatrisation et de fibrose cicatricielle et sur les conditions de charge (volémie, fonction vasculaire). Les médications anti-aldostérones peuvent antagoniser ces effets délétères mais elles ne sont pas dépourvues d’effets indésirables et on ne sait pas encore identifier les patients susceptibles d’en bénéficier réellement : l’hypertension artérielle pourrait être une condition préalable (étude EPHESUS), comme d’ailleurs l’atteinte d’un seuil critique de taille de l’infarctus (principal déterminant du remodelage). Notre hypothèse est que la relation entre aldostéronémie et événements cardiovasculaires pourrait être en grande partie expliquée par les effets délétères précoces et à long terme de l’aldostérone sur le remodelage cardiaque.

**L’objectif** **principal** sera de déterminer si l’aldostéronémie s’avère prédictive de l’apparition d’un remodelage dans les 6 mois suivant un infarctus avec sus-décalage du segment ST (STEMI) revascularisé en phase aiguë, indépendamment des paramètres prédictifs conventionnels (en particulier de la taille de l’infarctus, l’âge et hypertension artérielle).

**Les objectifs secondaires** seront d’individualiser des mécanismes et/ou des situations propices à l’action délétère de l’aldostérone sur le remodelage, en recherchant des associations entre d’une part, l’aldostéronémie et d’autre part, le fonctionnement vasculaire (rigidité aortique, résistances périphériques), les signes biologiques de fibrose et de remaniement matriciel et certaines situations cliniques (HTA, infarctus étendu, âge avancé…).

**Matériels et méthodes**. Une IRM cardio-vasculaire et des prélèvements sanguins seront réalisés entre les 2^ème^ et 4^ème^ jours après l’infarctus, puis 6 mois plus tard, chez 250 patients traités par revascularisation en phase aiguë d’un STEMI au CHU de Nancy. Au moins 40 patients pourraient avoir un remodelage délétère, ce qui permettrait d’étudier les influences combinées d’au moins 4 variables indépendantes telles que l’aldostéronémie, l’étendue de l’infarctus, l’âge et l’HTA.

Nous utiliserons la précision de l’IRM cardio-vasculaire : **(i)** pour évaluer puis suivre l’évolution de la fonction ventriculaire gauche après l’infarctus (volume, masse, fraction d’éjection), le remodelage étant défini par une augmentation de plus de 20 % du volume télédiastolique et **(ii)** pour quantifier des déterminants de ce remodelage, en particulier la taille de l’infarctus mais aussi des paramètres vasculaires intervenant sur les conditions de charge (résistances périphériques, vélocité de l’onde de pouls aortique). Les prélèvements sanguins permettront de mesurer : l’aldostéronémie, la réninémie, le BNP, la CRP ultrasensible et le procollagène de type 3, un marqueur de la fibrose collagène qui semble particulièrement impliqué dans le remodelage en post-infarctus et sur lequel l’aldostérone a une indéniable influence.

**La durée** prévue de l’étude, qui comprend la phase d’inclusion des patients et les examens à 6 mois, est de 2 ans. **Le budget** nécessaire est de 215 k€.

**Résultats attendus et perspectives**. Cette étude devrait permettre de mieux caractériser : **(i)** le remodelage cardiaque en post-infarctus et ses relations avec l’aldostéronémie, **(ii)** les mécanismes supposés de l’action délétère de l’aldostérone dans cette situation (signes biologiques de fibrose et de remaniement matriciel, paramètres intervenant sur les conditions de charge…) et **(iii)** les situations où l’aldostéronémie est réellement associée à un remodelage délétère et où les médications anti-aldostérone pourraient être particulièrement utiles (HTA, infarctus étendu, âge avancé…).

# Liste des abreviations

ACFA : Arythmie Complète par Fibrillation Auriculaire.

BNP : Brain Natriuretic Proteine

CPK-MB : Créatinine PhosphoKinase de type MB

CRP : C Reactive Proteine

ECG : Electro CardioGramme

HTA : HyperTension Artérielle

IEC : Inhibiteur de l’Enzyme de Conversion

IRM : Imagerie par Résonance Magnétique

PAI 1 : Inhibiteur des Activateurs du Plasminogène de type 1

PIII NP : Extrémité N terminale du Propeptide du Collagène de type III

SSFPS: Steady State Free Precession S

STEMI : Infarctus Myocardique avec sus décalage du segment ST

VOP : Vélocité de l’Onde de pouls aortique.

# Justification scientifique et description générale de la recherche

La prévalence de l’insuffisance cardiaque**,** et en particulier celle d’origine ischémique, augmente continuellement en France, comme d’ailleurs dans tous les pays industrialisés, ce qui constitue un véritable problème médico-économique (1). Après un infarctus myocardique, la survenue d’un **remodelage cardiaque délétère**, avec les majorations progressives de la dilatation et de l’hypokinésie du ventricule gauche, est la principale cause de cette évolution vers l’insuffisance cardiaque (2). La sévérité des séquelles initiales d’infarctus en est alors très certainement le déterminant principal (2-7), mais ce remodelage varie aussi en fonction d’autres paramètres. La prescription de certains médicaments est susceptible de le ralentir et c’est en particulier le cas des bêta-bloquants et surtout, des inhibiteurs de l’enzyme de conversion (IEC) dont la prescription est recommandée après un infarctus myocardique (2). A l’inverse, l’âge avancé est un facteur aggravant bien connu (2, 8), de même que l’hypertension artérielle, bien que ceci soit un peu plus discuté (2, 9, 10). Surtout, des études récentes suggèrent un effet délétère de **la sécrétion endogène d’aldostérone,** même chez les patients traités par IEC, mais sans que le mécanisme en soit bien compris (11-16). Les médications anti-aldostérone ont d’ailleurs un effet pronostique bénéfique lorsque l’infarctus est compliqué d’une insuffisance cardiaque, mais principalement lorsqu’il existe aussi un antécédent d’HTA (analyse en sous groupe de l’étude EPHESUS (17)). Cependant, l’aldostéronémie reste un index pronostique puissant lorsqu’elle est mesurée dans les toutes premières heures de l’infarctus et ceci, même en l’absence initiale d’insuffisance cardiaque et de dysfonction systolique sévère (11, 14). Ceci suggère des mécanismes liant l’aldostérone au remodelage (15, 16), mais ces mécanismes sont encore mal connus. C’est pourquoi les conditions optimales de l’utilisation des médicaments anti-aldostérone restent à définir dans cette situation clinique du post-infarctus.

**Dans cette étude nous avons le projet** **de caractériser le remodelage cardiaque en post-infarctus et ses relations avec l’aldostéronémie**, en recherchant des mécanismes et des situations propices à l’action délétère de l’aldostérone dans cette situation. Pour cela, nous utiliserons la capacité de l’IRM à réaliser une analyse multiparamètrique de ce remodelage, ainsi que des dosages de biomarqueurs du remodelage et en particulier de l’activité systémique de fibrose collagène.

## Aldostérone, infarctus myocardique et insuffisance cardiaque

L'hormone minéralocorticoïde aldostérone participe à la réabsorption du sodium par le rein et par conséquent, joue un rôle clé dans la **régulation du volume plasmatique et de la pression artérielle**. L'aldostérone exerce ses effets sur le tube rénal distal via un récepteur nucléaire qui agit comme un facteur de transcription dépendant des ligands (18). Il a été montré que ce récepteur est aussi exprimé par des cellules non épithéliales, telles que les cardiomyocytes et les cellules vasculaires endothéliales ou musculaires lisses, mais son rôle est alors beaucoup moins bien connu.

En fait, la stimulation de ces récepteurs rénaux et extra rénaux pourrait jouer un rôle dans des **maladies impliquant un remodelage cardiaque, vasculaire ou rénal**, indépendamment ou en synergie avec les effets de l'angiotensine 2 (19). En utilisant la cohorte Framingham, il a par exemple été montré qu'une augmentation des niveaux de l’aldostéronémie, même dans des proportions physiologiques, prédisposait au développement de l'hypertension artérielle (19). Un autre exemple est celui des patients avec hyperaldostéronisme primaire, qui associe une hypertension, une hypokaliémie, une élévation du rapport aldostéronémie/réninémie, et une hyperaldostéronémie. Chez ces patients, on observe une augmentation de la fréquence des évènements cardiovasculaires et ceci, bien au delà de ce qui peut être simplement expliqué par l’hypertension artérielle (20, 21). Enfin, dans la dernière décennie, le groupe de Nancy a beaucoup participé à la démonstration de l’influence pronostique péjorative de l’aldostérone dans l'insuffisance cardiaque. Cette démonstration a été obtenue par l’intermédiaire d’études cliniques ayant clairement montré l’effet bénéfique des médications anti-aldostérone chez les patients insuffisants cardiaques (études RALES et EPHESUS (12, 13, 17, 22)).

Comme dans l’insuffisance cardiaque, **l’infarctus myocardique** s’accompagne d’une activation neuro-hormonale délétère du système rénine-angiotensine-aldostérone ; et c’est une des raisons pour lesquelles la prescription d’IEC est recommandée dans cette situation (1). Cependant, chez les patients traités par IEC après un infarctus myocardique aigu, les dosages d’aldostéronémie restent fortement corrélés au risque de la survenue d’une complication grave et à celui de l’apparition d’une **insuffisance cardiaque** symptomatique (11, 14). Depuis plusieurs années, on savait déjà que l’aldostéronémie était anormalement élevée dans les infarctus les plus graves car les plus étendus. D’ailleurs, dans la situation où l’infarctus s’accompagne d’une insuffisance cardiaque en phase aiguë, l’étude EPHESUS a montré dès 2003 que la prescription d’un médicament bloquant les récepteurs minéralo-corticoides (l’éplérénone) avait un impact pronostique très favorable (12, 13).

Cependant, des études plus récentes ont montré que l’aldostéronémie, prélevée très tôt, dès l’admission des patients et avant même la revascularisation, restait un indice pronostique très puissant. A la différence de l’étude EPHESUS, ceci était alors observé dans des conditions où l’infarctus était beaucoup moins sévère, sans insuffisance cardiaque initiale, et alors que les valeurs de l’aldostéronémie étaient le plus souvent dans les limites des valeurs normales. Les patients dont l’aldostéronémie était située dans le quartile ou tercile supérieur avaient un risque nettement accru de décès et d’évolution vers l’insuffisance cardiaque, aussi bien à court terme qu’à long terme (11, 14), et le remodelage délétère pourrait en être un mécanisme essentiel.

En effet, cette **relation entre aldostérone et remodelage** a déjà été démontrée dans des études réalisées chez des patients revascularisés en phase aiguë d’un infarctus myocardique (14, 15). Ces patients étaient randomisés entre un traitement associant ou non une médication anti-aldostérone après la reperfusion, et la prescription de cette médication était associée à des diminutions : (i) de l’aldostéronémie et de l’extraction myocardique de l’aldostérone et (ii) du remodelage ultérieur (moindre augmentation du volume télé-diastolique, meilleure récupération de la fraction d’éjection).

**Cependant, on** **ne connaît pas encore précisément le mécanisme de cet effet favorable**, **de même que les catégories de patients susceptibles d’en bénéficier réellement**. Dans l’étude EPHESUS, par exemple, l’effet favorable de l’éplérénone sur la mortalité n’a pu être clairement démontré qu’en présence d’un antécédent d’hypertension artérielle (17). Ceci suggère des mécanismes d’action spécifique chez ces patients. On peut par ailleurs supposer que les médications anti-aldostérone auront peu d’effet favorable lorsque l’infarctus est limité, en particulier chez les patients jeunes. En effet, l’âge avancé (2, 8) et surtout la taille initiale de l’infarctus (2-7) sont deux déterminants essentiels du remodelage en post-infarctus. Les médications anti-aldostérones ne sont d’ailleurs pas dénuées d’effets indésirables, en particulier lorsqu’elles sont associées aux autres médications du post-infarctus (IEC, diurétiques..). Il semble donc particulièrement utile de pouvoir identifier les situations où l’aldostérone est responsable d’une aggravation du remodelage en post-infarctus et aussi, de préciser les mécanismes de cette action délétère.

Plusieurs **actions biologiques de l’aldostérone** pourraient être directement ou indirectement impliquées :

**(i)** Il peut tout d’abord s’agir d’actions cardiaques directes telles que la stimulation de l’hypertrophie pariétale et surtout de la fibrose tissulaire myocardique, et ceci a déjà été bien caractérisé chez l’animal (23). Chez l’homme, l’hypothèse d’une stimulation excessive de la fibrose myocardique est surtout soutenue par des arguments indirects, issus de la mesure de biomarqueurs sanguins. Les médications anti-aldostérone ont en effet la propriété de diminuer les concentrations sanguines en certains marqueurs plasmatiques de la fibrose collagène et en particulier du PIIINP. Par exemple, chez les patients revascularisés en phase aiguë d’un infarctus antérieur, une diminution du PIIINP est associée à l’action favorable de ces médicaments sur le remodelage (15, 16). Dans une autre situation clinique, qui est celle des patients insuffisants cardiaques chroniques, la prescription d’un traitement anti-aldostérone était associée à des diminutions conjointes du PIIINP et de l’incidence des complications graves (étude RALES (22)).

**(ii)** Un autre mécanisme potentiel est celui de l’action rénale bien connue de l’aldostérone avec, en particulier, la rétention urinaire du sodium et les excrétions de potassium et magnésium. Ces effets pourraient avoir des conséquences sur les risques de trouble du rythme grave (24), ainsi que sur la volémie et donc, sur les conditions de charge du fonctionnement cardiaque.

**(iii)** Enfin, l’aldostérone pourrait indirectement nuire au fonctionnement cardiaque par l’intermédiaire d’atteintes vasculaires artérielles puisque l’aldostérone est susceptible d’accroître le tonus vasculaire, les résistances périphériques et la rigidité artérielle à court ou à long terme par divers mécanismes (dysfonction endothéliale, PAI 1, inflammation, fibrose tissulaire…) (25-29).

**Ainsi, l’aldostérone est susceptible** **d’interagir à la fois sur les mécanismes de cicatrisation et sur les conditions de charge** du fonctionnement cardiaque (volémie, fonction vasculaire). Pour pouvoir analyser conjointement ces actions délétères multiples, **notre étude sera multiparamètrique** avec : **(i)** **l’utilisation de l’IRM** pour permettre une évaluation fonctionnelle précise non seulement cardiaque mais aussi vasculaire et **(ii)** **l’association à des dosages sériques** permettant de suivre les évolutions de certains biomarqueurs en particulier ceux témoignant de la fibrose tissulaire.

## L’IRM pour quantifier les séquelles d’infarctus, le remodelage cardiaque et le fonctionnement vasculaire

**L’IRM cardiaque** est une technique de développement récent et qui s’avère particulièrement informative dans le domaine du remodelage cardiaque en post-infarctus, en raison de sa capacité à quantifier : **(i)** les variations de la structure et du fonctionnement cardiaques, qui caractérisent le remodelage cardiaque à l’échelle macroscopique (dilatation, hypertrophie, hypokinésie) et **(ii)** l’étendue des séquelles d’infarctus et leur sévérité en terme d’extension transmurale, car il s’agit du principal déterminant du remodelage cardiaque (Figure 1).

**Déterminer la sévérité d’un infarctus** permet en effet de prédire au moins en partie le remodelage cardiaque ischémique et cette détermination est d’autant plus importante que la sévérité des infarctus s’avère extrêmement variable**,** aussi bien en pratique clinique, que sur les modèles d’infarctus expérimentaux réalisés chez l’animal (2-8). Cette variabilité concerne, non seulement l’étendue en surface de la zone infarcie, mais aussi la sévérité et l’importance des destructions cellulaires dans l’épaisseur pariétale. En effet, ce sont surtout les zones d’infarctus transmural ou quasi transmural, c’est-à-dire celles touchant au moins 50 à 70% du pool cellulaire initial, qui sont susceptibles d’entraîner un remodelage cardiaque délétère (3, 5, 30-32). Dans ces conditions, les territoires infarcis évoluent vers un amincissement avec expansion pariétale, ce qui est lié à un phénomène de « glissement » entre cardiomyocytes voisins (31). Ce glissement peut être expliqué par l’augmentation des contraintes pariétales et par la déstructuration de la matrice extracellulaire sous l’action des métallo-protéinases activées (1, 31, 32). L’évolution est alors fréquemment celle de la dilatation anévrismale dont la signification pronostique est particulièrement péjorative.


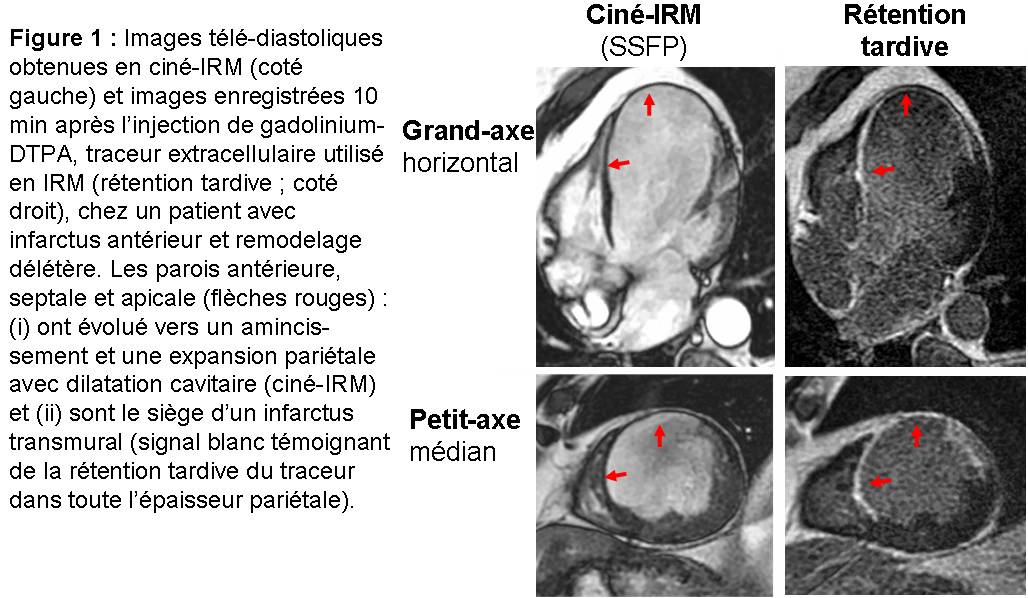


**L’IRM est la seule technique d’imagerie médicale qui permette d’évaluer l’extension transmurale de l’infarctus** et ceci, en utilisant des traceurs qui ont la propriété d’être retenus tardivement dans les territoires infarcis. Cette technique dite de « rétention tardive » a déjà fait l’objet de plusieurs études dans notre équipe (33-36). Elle s’avère particulièrement efficace pour différencier les territoires nécrosés de ceux viables et dont la dysfonction est liée à une sidération ou à une hibernation myocardique.

Surtout, des études récentes ont permis de montrer que lorsqu’elle était évaluée en IRM dans les 2 à 3 jours suivant un infarctus reperfusé (5, 6)**, la sévérité des séquelles d’infarctus était un paramètre prédictif très puissant: (i)** **du risque de la survenue de complications ultérieures** et en particulier, du risque d’hospitalisation pour insuffisance cardiaque et **(ii) du remodelage ultérieur,** qui était alors caractérisé en IRM par une augmentation de plus de 20 % du volume télédiastolique à 4 mois (5).

Ce critère d’une augmentation supérieure à 20 % a initialement été décrit dans des études d’échographie (37) et il définit un seuil au delà duquel le remodelage a une réelle signification pronostique péjorative (5, 30, 37). Il s’agit cependant d’un domaine où l’IRM pourrait être nettement supérieure à l’échographie, car cet examen est particulièrement précis et sensible pour détecter les **variations du volume ventriculaire gauche** (38), comme d’ailleurs celles de la fraction d’éjection ou de la masse ventriculaire gauche, en particulier lorsqu’on utilise les nouvelles séquences de précession à l’équilibre (Figure 2). On sait par exemple que l’inclusion de 10 patients peut être suffisante pour que l’IRM puisse mettre en évidence une variation spontanée ou post thérapeutique de 10 ml du volume télédiastolique ou de 10 g pour la masse myocardique ; alors qu’en échographie, qui est beaucoup moins reproductible, les nombres de patients nécessaires sont beaucoup plus importants : 53 et 190, respectivement (38, 39).


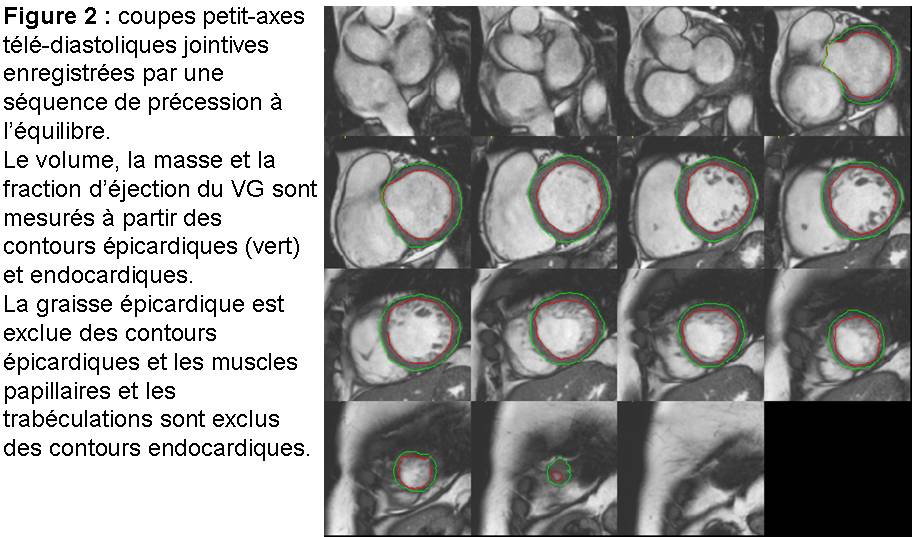


**L’IRM permet aussi d’analyser le fonctionnement vasculaire** grâce à des séquences de « contraste de phase », qui permettent de mesurer très précisément des débits sanguins, en particulier lorsqu’il s’agit de flux artériels non turbulents et que les vaisseaux ont un calibre suffisant. C’est, par exemple, le cas des mesures faites à la racine de l’aorte et qui permettent d’évaluer le débit cardiaque total, ainsi que les **résistances artérielles périphériques totales** (débit cardiaque / pression artérielle systémique moyenne) (40, 41).

Ces séquences de contraste de phase permettent aussi de mesurer la **vélocité de l’onde de pouls aortique** (VOP) (41-44). Comme cela est illustré en figure 3, cette technique consiste à mesurer l’intervalle de temps séparant le début de l’onde de flux aortique, entre un site proximal et un site distal, puis à diviser la longueur artérielle séparant ces 2 sites par l’intervalle de temps mesuré. Lorsque les parois artérielles sont normalement élastiques, l’arrivée du flux pulsé aortique s’accompagne d’une dilatation de l’aorte, ce qui ralentit la VOP et prévient la survenue de valeurs excessives de pression pulsée (45). L’augmentation de cette vitesse témoigne donc d’une majoration de la rigidité aortique et elle s’accompagne aussi d’une augmentation des conditions de charge, en raison de modifications touchant les ondes de réflexion (augmentation d’amplitude et survenue plus précoce, avant la fin de la phase de contraction systolique (46-48)). Nous avons récemment développé la mesure de la VOP en IRM, ce qui nous a permis de montrer que ce paramètre était un déterminant de la masse myocardique à un stade précoce et encore non compliqué de l’obésité abdominale. Ceci suggère qu’une augmentation ultérieure de la rigidité aortique pouvait être une des causes du remodelage cardiaque des patients obèses (41).


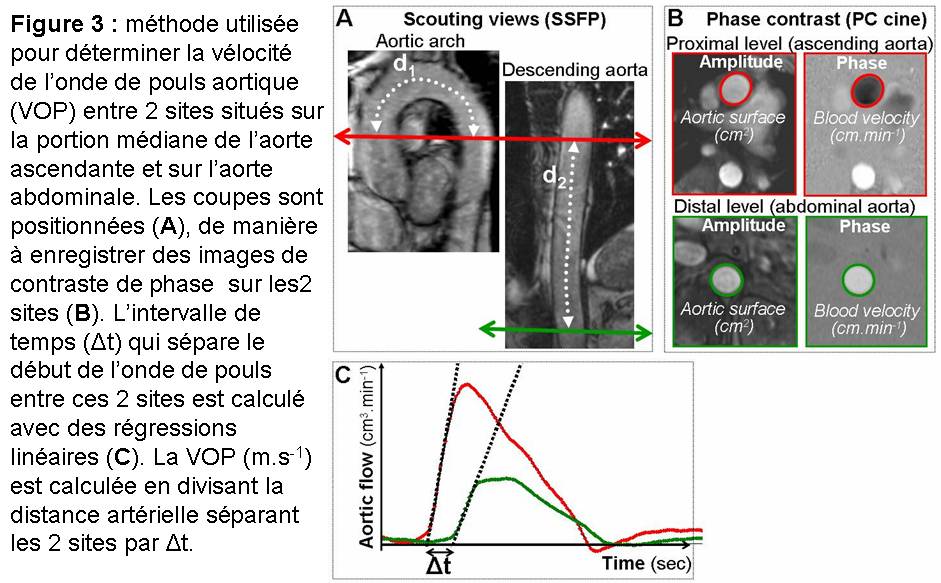


**L’IRM vasculaire offre donc l’opportunité de suivre l’évolution de la rigidité aortique et des résistances vasculaires systémiques, deux paramètres susceptibles** : **(i)** d’être modulés par les multiples actions vasculaires de l’aldostérone et **(ii)** de faire varier les conditions de charge et la tension pariétale des zones infarcies et donc, d’interagir directement sur le remodelage.

## Biomarqueurs plasmatiques ET ACTIVITE de fibrose SYSTEMIQUE

La matrice extracellulaire cardiaque est essentiellement composée de collagène de type I et III qui sont synthétisés à partir du clivage de propeptides spécifiques. Les dosages sériques des produits de clivage N-terminaux de ces propeptides s’avèrent particulièrement intéressants en recherche clinique puisqu’ils reflètent l’importance de la synthèse collagène dans l’organisme. Surtout, plusieurs études ont déjà montré l’intérêt du dosage de **l’extrémité N-terminale du propeptide du collagène de type III (PIIINP)** chez les patients victimes d’un infarctus (15, 16, 49-51). On observe en effet normalement une augmentation des concentrations sériques en PIIINP dans les jours suivant l’infarctus, ce qui témoigne du processus normal de réparation et de cicatrisation. Cependant, l’importance et surtout la persistance dans le temps de cette augmentation de concentration sont associées à une détérioration du pronostic et à une majoration du remodelage délétère, témoignant alors d’une fibrose myocardique excessive.

Dans notre étude, des dosages de PIIINP seront réalisés lors du bilan initial et lors du contrôle à 6 mois car ce paramètre est fortement influencé par l’aldostérone. En particulier, on peut rappeler qu’on sait déjà que les médications anti-aldostérone permettent de diminuer les concentrations sériques en PIIINP et que ceci était alors associé à un moindre remodelage (études en post-infarctus (15, 16)) et à une amélioration du pronostic (études RALES dans l’insuffisance cardiaque chronique (19)).

En outre, le PIIINP pourrait aussi refléter l’importance de la rigidité aortique, comme cela a déjà été montré chez des patients insuffisants cardiaques chroniques (52). Rappelons que notre étude permettra d’analyser aussi la rigidité aortique, qui pourrait être un facteur favorisant du remodelage cardiaque délétère (augmentation des conditions de charge du fonctionnement cardiaque).

D’autres biomarqueurs, qui seront mesurés dans notre étude, sont susceptibles de donner des informations pronostiques et surtout cognitives et mécanistiques dans ce domaine du post-infarctus. Il s’agit en particulier du Brain Natriuretic Peptid (BNP) (53), qui est un index de dysfonction cardiaque et surtout de tension pariétale, et de la C Reactive Proteine (CRP) ultra-sensible, qui renseigne sur l’intensité de la réaction inflammatoire.

Il sera aussi réalisé des dosages : (i) de la réninémie, permettant de calculer le rapport aldostéronémie/réninémie, moins influencé par les traitements médicamenteux que l’aldostéronémie mesurée isolément, et qui reflète l’importance de la stimulation du système rénine-angiotensine, et (ii) de la créatininémie, permettant d’estimer le débit de filtration glomérulaire (formule MDRD), prédicteur indépendant du remodelage et du pronostic cardiovasculaires (54), et paramètre corrélé au degré d’activation du système rénine-angiotensine (par exemple, une insuffisance rénale sévère s’accompagne habituellement d’un hyporéninisme hypoaldostéronisme, secondaire à une rétention hydrosodée).

# Objectifs de la recherche

## Objectif principal

**L’objectif principal** sera de déterminer si l’aldostéronémie s’avère prédictive de l’apparition d’un remodelage dans les 6 mois suivant un STEMI (infarctus myocardique avec sus-décalage du segment ST) revascularisé en phase aiguë, indépendamment des paramètres prédictifs conventionnels (taille de l’infarctus mesurée en IRM, âge, HTA…).

## Objectif secondaire

**L’objectif secondaire** sera d’individualiser des mécanismes potentiels et/ou des situations propices à l’action délétère de l’aldostérone sur le remodelage en recherchant des associations entre, d’une part l’aldostéronémie, et d’autre part les paramètres témoignant : (i) du fonctionnement vasculaire, (ii) du fonctionnement cardiaque, (iii) des signes biologiques de fibrose, d’inflammation systémique et de tension pariétale excessive, de la fonction rénale et (iii) de certaines situations cliniques (HTA, infarctus étendu, âge avancé…).

## Critère principal de jugement

Associations entre, d’une part le remodelage cardiaque délétère (défini comme une augmentation de plus de 20% du volume télédiastolique du ventricule gauche), et d’autre part l’aldostéronémie mesurée lors du bilan initial et à 6 mois, indépendamment des autres déterminants du remodelage (en particulier, de la taille de l’infarctus, de l’âge et des antécédents d’HTA).

## Critères secondaires de jugement

Associations entre d’une part, les aldostéronémies mesurées lors du bilan initial et à 6 mois (et les variations entre ces 2 valeurs) et d’autre part :

- les paramètres mesurés lors du bilan initial et à 6 mois et témoignant : (i) du fonctionnement vasculaire (pression artérielle, rigidité aortique et résistances périphériques mesurées en IRM), (ii) du fonctionnement cardiaque (débit et index cardiaque, volumes télésystolique et télédiastolique du ventricule gauche, fraction d’éjection du ventricule gauche, étendue de l’infarctus) et (iii) de l’activité du système rénine angiotensine-aldostérone, de fibrose, de l’inflammation systémique, de la tension pariétale et de la fonction rénale (rapport aldostérone/rénine, concentrations plasmatiques de PIIINP, CRP ultra sensible, BNP, rénine, créatininémie et clairance de la créatinine selon la formule MDRD),
- certaines situations cliniques (HTA, infarctus étendu, âge avancé…).

# Conception de la recherche

Il s’agit d’une étude prospective, non contrôlée, non randomisée, longitudinale, en ouvert, monocentrique (CHU de Nancy).

L’inclusion de 250 patients serait nécessaire pour valider l’objectif principal du protocole (voir chapitre 10). L’IRM 3T utilisée pour cette étude a été financée partiellement par le dernier Contrat de Plan Etat Région, ce qui a permis de réserver 40% du temps d’utilisation de cet appareil à l’activité de recherche.

## Déroulement de l’étude

Les patients inclus seront ceux hospitalisés dans notre CHU pour un **primo-infarctus myocardique** **avec sus décalage du segment ST revascularisé en phase aiguë** par angioplastie et datant de moins de 4 jours. L’infarctus myocardique aigu avec sus décalage du segment ST (STEMI) sera défini par la présence des critères suivants : douleur thoracique prolongée, augmentation des enzymes cardiaques (CPK-MB > 2 fois la limite supérieure de la normale), et présence d’un sus décalage du segment ST d’au moins 2 mm et touchant au moins deux dérivations contiguës de l’ECG.

**Les critères d’exclusion** seront les suivants : (i) contre-indication à l’examen IRM (pace-maker, clip métallique neurochirurgical, claustrophobie sévère…) et/ou à l’injection de produit de contraste (antécédents d’hypersensibilité aux sels de gadolinium), (ii) état clinique instable (signes d’insuffisance cardiaque ou coronaire instable, HTA mal stabilisée sous traitement), (iii) rythme cardiaque non sinusal et (iv) mineurs, patients sous tutelle et patients n’ayant pas compris et/ou signé le formulaire de consentement éclairé.

Un investigateur présentera l’étude au patient. La note d’information spécifique à l’étude lui sera remise. Si le patient accepte de participer à l’étude, il lui sera demandé de parapher et de signer le formulaire de consentement éclairé en 3 exemplaires (voir paragraphe Error: Reference source not found).

**L’étude comprendra la réalisation d’un bilan initial et d’un bilan de suivi** qui seront tous deux réalisés dans les locaux de l’IRM 3T, à l’Hôpital Brabois : (i) dans les 2 à 4 premiers jours suivant le début des douleurs, pour le bilan initial, et (ii) 6 mois plus tard (± 15 jours) pour le bilan de suivi. Ces 2 bilans seront identiques et comprendront consécutivement :

- des prélèvements sanguins destinés à mesurer les différents biomarqueurs (volume = 30ml) (cf 5.2.2)
- une IRM cardio-vasculaire avec injection intraveineuse d’un produit de contraste (Dotarem ^TM^) (cf. 5.2.1) et mesures répétées de la pression artérielle systémique.

## Description des examens de l’étude

### IRM Cardio-vasculaire

L’examen sera réalisé sur un aimant de 3T (GE Medical Systems) avec une antenne 8 canaux, le patient étant en décubitus dorsal, équipé d’électrodes ECG pour la synchronisation des acquisitions et d’un brassard pour la mesure automatisée de la pression artérielle brachiale. La procédure d’examen comprendra : **1)** l’installation du patient et l’acquisition des images initiales de repérage (10 min), **2)** une analyse de la fonction ventriculaire gauche par une méthode conventionnelle utilisant une séquence de précession à l’équilibre (SSFPS) et des coupes petit-axes jointives (15 min (33, 34, 36, 55)), **3)** une mesure du débit cardiaque par une séquence de contraste de phase appliquée à la portion médiane de l’aorte ascendante (5 min ; (40, 41)), **4)** une mesure de la vélocité de l’onde pouls aortique entre un site proximal (portion médiane de l’aorte ascendante) et distal (portion proximale de l’aorte abdominale), avec des séquences de contraste de phase (5 min ;(41-44)) et **5)** l’injection intraveineuse d’un produit de contraste (Dotarem^TM^) avec analyse de la rétention tardive dans les 7 à 15 min suivantes (33-36).

Les valeurs des pressions artérielles systolique, diastolique et moyenne seront enregistrées automatiquement toutes les 10 minutes et les valeurs médianes de ces mesures seront utilisées pour les analyses ultérieures.

Tous les paramètres d’IRM cardiovasculaire seront mesurés par les 2 mêmes observateurs expérimentés. Une deuxième analyse consensuelle sera systématiquement réalisée lorsque ces mesures seront considérées comme étant éloignées et ce sont alors les nouvelles valeurs consensuelles qui seront utilisées dans les analyses ultérieures. Dans le cas contraire, ce sont les moyennes des 2 mesures qui seront utilisées.

#### Evaluation des volumes, masse et fraction d’éjection du ventricule gauche

Cette évaluation sera réalisée avec une séquence de précession à l’équilibre (FIESTA, GE Medical Systems) selon une procédure conventionnelle que nous avons déjà utilisée et publiée (33, 34, 36, 55). Cette procédure consiste en l’enregistrement, lors d’apnées de 10 à 15 sec, de coupes petit axes jointives couvrant l’ensemble du ventricule gauche et avec les paramètres suivants : 8 mm d’épaisseur de coupe, 45° d’angle de bascule, TE/TR d’environ 3,5/1,8 ms, 1 seule excitation, bande passante de 125 kHz, FOV de 32 à 36 mm, matrice 224x224 interpolée à 512x512, 1,5 de facteur d’accélération ASSET, enregistrement de 14 à 16 lignes par segment, 30 phases par cycle cardiaque avec interpolation..

Comme lors nos précédentes études (33, 34, 36, 55), les paramètres de la fonction ventriculaire gauche (masse, volume télésystolique, volume télédiastolique et fraction d’éjection) seront obtenus avec un logiciel spécifique (MASS™, Medis, Hollande). Avec celui-ci, les contours épicardiques et endocardiques peuvent être générés automatiquement sur les images télésystoliques et télédiastoliques, mais ils doivent ensuite être corrigés manuellement (Figure 2).

#### Mesure du débit cardiaque et des résistances périphériques totales

Cette mesure sera réalisée avec une séquence de contraste de phase (Fast PC Cine, GE Medical Systems) (40, 41) sur une coupe positionnée perpendiculairement à l’aorte ascendante et passant dans sa partie médiane. Cette coupe sera enregistrée en apnée et avec les paramètres suivants : 10 mm d’épaisseur de coupe, 15° d’angle de bascule, TE/TR d’environ 7/3 ms, 1 seule excitation, bande passante de 31 kHz, FOV de 32 à 36 mm, matrice 256x128, enregistrement de 6 lignes par segment, 32 phases par cycle cardiaque avec interpolation, codage unidirectionnel de la vitesse avec 150 cm/sec de vélocité maximale.

Le débit cardiaque (L.min^-1^) sera mesuré à partir des images de phase et d’amplitude de l’aorte ascendante avec un logiciel automatisé (« CV flow », Medis, Hollande). Ce débit sera aussi exprimé par m^2^ de surface corporelle (index cardiaque).

Les résistances vasculaires périphériques totales seront ensuite estimées par le rapport de la pression artérielle moyenne sur le débit cardiaque. Elles seront donc exprimées en mmHg.min.L^-1^ et par m^2^ de surface corporelle.

#### Mesure de la vélocité de l’onde de pouls aortique

Cette technique, que nous avons déjà utilisée dans une précédente étude (41) et qui a aussi été utilisée par d’autres équipes (42-44), consiste à mesurer l’intervalle de temps séparant le début de l’onde de flux aortique, entre un site proximal et un site distal, puis à diviser la longueur artérielle séparant ces 2 sites par l’intervalle de temps mesuré.

La longueur artérielle séparant les 2 sites sera mesurée avec une technique manuelle sur les images d’une acquisition multi-coupes tridimensionnelle couvrant l’ensemble du volume aortique (aorte thoracique et partie initiale de l’aorte abdominale) et qui sera préalablement enregistrée en respiration libre et avec une séquence d’écho de gradient conventionnelle.

La mesure de l’intervalle de temps entre les 2 sites sera réalisée avec la même séquence de contraste de phase que celle utilisée pour la mesure du débit cardiaque (Fast PC Cine, GE Medical Systems), mais avec une résolution temporelle plus élevée (7 ms) et un temps d’acquisition prolongé (2 min), l’enregistrement étant alors réalisé en respiration spontanée.

Comme cela est illustré en figure 3, 2 coupes axiales distantes de 15 cm seront positionnées de manière à ce que la première croise la partie médiane de l’aorte ascendante (site proximal), la deuxième croisant alors la portion initiale de l’aorte abdominale (site distal). Les paramètres d’enregistrement seront les suivants : 15 mm d’épaisseur de coupe, 15° d’angle de bascule, TE/TR d’environ 7/3 ms, 1 seule excitation, bande passante de 31 kHz, FOV de 32 à 36 mm, matrice 256x128, enregistrement de une ligne par segment, 200 phases par cycle cardiaque avec interpolation, codage unidirectionnel de la vitesse avec 150 cm/sec de vélocité maximale.

#### Injection de Dotarem^TM^ et enregistrement des images de rétention tardive

Une dose de 0,1 mmol/kg de Dotarem^TM^ (Laboratoire Guerbet, France) sera injectée en bolus par voie intra-veineuse. Comme dans une de nos dernières études (33), les images de rétention tardive seront enregistrées entre la 8^ème^ et la 14^ème^ minutes avec une séquence tridimensionnelle multi coupes d’écho de gradient segmenté comprenant une impulsion d’inversion préparatoire et permettant une synchronisation de l’enregistrement en télédiastole (3D-MDE, GE Medical Systems). Cette séquence permet de couvrir la quasi-totalité du volume ventriculaire gauche en enregistrant de 10 à 15 coupes jointives de 10 mm d’épaisseur chacune et ceci, en une seule apnée d’une durée de 15 à 20 secondes. Un minimum de 3 acquisitions volumiques de ce type seront réalisées dans les 3 orientations petit-axe, grand-axe vertical et grand-axe horizontal, et avec les paramètres suivants : temps d’inversion modulé entre 180 et 280 ms de manière à annuler le signal myocardique, 8 mm d’épaisseur de coupe, FOV de 30 à 36 cm, matrice 224x224, 2 de facteur d’accélération ASSET, 15° d’angle de bascule, TE/TR d’environ 4,5/2 ms, 1 seule excitation, bande passante de 61,5 kHz.

La taille de l’infarctus sera déterminée selon 2 manières qui se sont déjà révélées très efficaces pour prédire le remodelage ventriculaire gauche en post-infarctus (5, 6) :

- Une première méthode utilise un logiciel spécifique (MASS™, Medis, Hollande) d’aide à la délimitation des zones de rétention tardive sur les coupes petit-axes jointives (5). Une correction manuelle est cependant nécessaire, en particulier pour inclure les zones d’obstruction micro-vasculaire (zones d’absence de signal, sans contraste, entourées par des zones de rétention tardive). Le volume infarci est ensuite exprimé en valeur absolue, ainsi qu’en pourcentage du volume ventriculaire gauche (5).
- Une deuxième méthode utilisera la technique de segmentation du ventricule gauche en 17 parties avec l’analyse visuelle : (i) de 3 coupes petit-axes respectivement située au milieu des tiers basal, médian et apical du ventricule gauche et (ii) des coupes grand-axes médio-ventriculaires, qui permettent d’analyser plus précisément le 17^ème^ segment apical (6). Le volume d’infarctus transmural sera alors estimé de manière relative, selon le pourcentage de segments présentant une rétention transmurale du traceur (définie par une extension sur au moins 75 % de l’épaisseur pariétale) ou bien les signes d’obstruction micro-vasculaire dont l’aspect a déjà été défini dans le précédent paragraphe.

### Biomarqueurs

Les biomarqueurs, dont les concentrations plasmatiques seront mesurées lors du bilan initial et à 6 mois, sont susceptibles de donner des informations sur le remodelage ventriculaire gauche. Il s’agit de l’aldostéronémie mais aussi de marqueurs sériques : 1) d’inflammation (CRP ultra-sensible), 2) de dysfonction et de tension pariétale cardiaque (BNP), 3) de fibrose collagène (procollagène de type 3). Il sera aussi réalisé des dosages : (i) de la réninémie, permettant de calculer le rapport aldostérone/rénine, et (ii) de la créatininémie, permettant d’estimer le débit de filtration glomérulaire par la formule MDRD (56) :

eGFR [mL/min/l.73 m²] = 186 × (Serum créatinine [mg/dL])^-1.154^ × (Age [y])^-0.203^ (× 0.742 if female)

Le prélèvement veineux sera recueilli dans 6 tubes : 2 tubes héparinés (héparinate de lithium) de 4 ml chacun, 2 tubes sec avec gel séparateur de 4 ml et 2 tubes de 7 ml EDTA. Les tubes seront rapidement centrifugés à température ambiante pendant 10 minutes à 3000 g, le surnageant (plasma ou sérum) étant ensuite conservé à -80°C.

#### Aldostéronémie et réninémie

L’aldostéronémie sera mesurée par RIA (kit Siemens TKAL2, limite de détection 11pg/L) et la réninémie, sera aussi mesurée par RIA (kit CIS BIO, limite de détection  < 1pg/L).

#### Marqueur sérique de fibrose collagène

Nous mesurerons un marqueur de synthèse du collagène de type III, le peptide amino-terminal du procollagène de type III [PIIINP], à l’aide d’un kit de radioimmunologie commercialisé par Orion Diagnostica (Espoo, Finlande). La limite de détection est de: 0,3 μg/L.

#### Marqueur sérique d’inflammation

La CRP ultrasensible sera mesurée par immuno-turbidimétrie (limite de détection : 1,2 mg/L).

#### Marqueur sérique de dysfonction et de tension pariétale cardiaque

Le BNP sera mesurée par immunoluminescence (limite de détection 1 pg/mL).

#### Créatininémie

La créatinine sanguine sera mesurée par méthode cinétique photométrique (limite de détection

10 μmol/L).

**REFERENCES**

1. Roger VL. Coronary disease surveillance: a public health imperative. Eur. Heart J. 2007; 28(17): 2051 - 2052.
2. Cohn JN, Ferrari R, Sharpe N. Cardiac remodeling-concepts and clinical implications: a consensus paper from an international forum on cardiac remodeling. Behalf of an International Forum on Cardiac Remodeling. J Am Coll Cardiol 2000;35(3):569-82.
3. Maskali F, Franken PR, Poussier S, Tran N, Vanhove C, Boutley H, Le Gall H, Karcher G, Zannad F, Lacolley P, Marie PY. Initial infarct size predicts subsequent cardiac remodeling in the rat infarct model: an in vivo serial pinhole gated SPECT study. J Nucl Med 2006;47(2):337-44.
4. Savoye C, Equine O, Tricot O, Nugue O, Segrestin B, Sautiere K, Elkohen M, Pretorian EM, Taghipour K, Philias A, Aumegeat V, Decoulx E, Ennezat PV, Bauters C; REmodelage VEntriculaire study group. Left ventricular remodeling after anterior wall acute myocardial infarction in modern clinical practice (from the REmodelage VEntriculaire [REVE] study group). Am J Cardiol 2006;98(9):1144-9.
5. Wu E, Ortiz JT, Tejedor P, Lee DC, Bucciarelli-Ducci C, Kansal P, Carr JC, Holly TA, Lloyd-Jones D, Klocke FJ, Bonow RO. Infarct size by contrast enhanced cardiac magnetic resonance is a stronger predictor of outcomes than left ventricular ejection fraction or end-systolic volume index: prospective cohort study. Heart. 2008;94(6):730-6.
6. Tarantini G, Razzolini R, Cacciavillani L, Bilato C, Sarais C, Corbetti F, Marra MP, Napodano M, Ramondo A, Iliceto S. Influence of transmurality, infarct size, and severe microvascular obstruction on left ventricular remodeling and function after primary coronary angioplasty. Am J Cardiol 2006;98(8):1033-40.
7. Rubenstein JC, Ortiz JT, Wu E, Kadish A, Passman R, Bonow RO, Goldberger JJ. The use of periinfarct contrast-enhanced cardiac magnetic resonance imaging for the prediction of late postmyocardial infarction ventricular dysfunction. Am Heart J. 2008;156(3):498-505.
8. Mahara K, Anzai T, Yoshikawa T, Maekawa Y, Okabe T, Asakura Y, Satoh T, Mitamura H, Suzuki M, Murayama A, Ogawa S; Keio Interhospital Cardiology Study (KICS) investigators. Aging adversely affects postinfarction inflammatory response and early left ventricular remodeling after reperfused acute anterior myocardial infarction. Cardiology. 2006;105(2):67-74.
9. Parodi G, Carrabba N, Santoro GM, Memisha G, Valenti R, Buonamici P, Dovellini EV, Antoniucci D. Heart failure and left ventricular remodeling after reperfused acute myocardial infarction in patients with hypertension. Hypertension. 2006;47(4):706-10.
10. Kenchaiah S, Pfeffer MA, St John Sutton M, Plappert T, Rouleau JL, Lamas GA, Sasson Z, Parker JO, Geltman EM, Solomon SD. Effect of antecedent systemic hypertension on subsequent left ventricular dilation after acute myocardial infarction (from the Survival and Ventricular Enlargement trial). Am J Cardiol. 2004; 94: 1–8.
11. Palmer BR, Pilbrow AP, Frampton CM, Yandle TG, Skelton L, Nicholls MG, Richards AM. Plasma aldosterone levels during hospitalization are predictive of survival post-myocardial infarction. Eur Heart J. 2008; 29(20):2489-96.
12. Pitt B, Remme W, Zannad F, Neaton J, Martinez F, Roniker B, Bittman R, Hurley S, Kleiman J, Gatlin M. Eplerenone, a selective aldosterone blocker, in patients with left ventricular dysfunction after myocardial infarction. N Engl J Med 2003; 348:1309–1321.
13. Pitt B, Zannad F, Remme WJ, Cody R, Castaigne A, Perez A, Palensky J, Wittes J. The effect of spironolactone on morbidity and mortality in patients with severe heart failure. Randomized Aldactone Evaluation Study Investigators. N Engl J Med 1999; 341:709–717.
14. Beygui F, Collet JP, Benoliel JJ, Vignolles N, Dumaine R, Barthelemy O, Montalescot G. High plasma aldosterone levels on admission are associated with death in patients presenting with acute ST-elevation myocardial infarction. Circulation 2006; 114:2604–2610.
15. Modena MG, Aveta P, Menozzi A, Rossi R. Aldosterone inhibition limits collagen synthesis and progressive left ventricular enlargement after anterior myocardial infarction. Am Heart J 2001 ; 141:41–46.
16. Hayashi M, Tsutamoto T, Wada A, Tsutsui T, Ishii C, Ohno K, Fujii M, Taniguchi A, Hamatani T, Nozato Y, Kataoka K, Morigami N, Ohnishi M, Kinoshita M, Horie M. Immediate administration of mineralocorticoid receptor antagonist spironolactone prevents post-infarct left ventricular remodeling associated with suppression of a marker of myocardial collagen synthesis in patients with first anterior acute myocardial infarction. Circulation 2003; 107:2559–2565
17. Pitt B, Ahmed A, Love TE, Krum H, Nicolau J, Cardoso JS, Parkhomenko A, Aschermann M, Corbalán R, Solomon H, Shi H, Zannad F. History of hypertension and eplerenone in patients with acute myocardial infarction complicated by heart failure. Hypertension. 2008; 52(2):271-8.
18. Farman N. Molecular and cellular determinants of mineralocorticoid selectivity. Curr Opin Nephrol Hypertens. 1999;8(1):45-51.
19. Weber KT. Aldosterone in congestive heart failure. N Engl J Med. 2001 ; 345(23):1689-97.
20. Milliez P, Girerd X, Plouin PF, Blacher J, Safar ME, Mourad JJ. Evidence for an increased rate of cardiovascular events in patients with primary aldosteronism. J Am Coll Cardiol. 2005;45(8):1243-8.
21. Plouin PF, Rossignol P, Amar L. Selection of patients for surgery for primary aldosteronism. Clin Exp Pharmacol Physiol. 2008;35:522-5
22. Zannad F, Alla F, Dousset B, Perez A, Pitt B. Limitation of excessive extracellular matrix turnover may contribute to survival benefit of spironolactone therapy in patients with congestive heart failure: insights from the randomized aldactone evaluation study (RALES). Rales Investigators. Circulation. 2000; 102(22):2700-6.
23. Young MJ, Lam EY, Rickard AJ. Mineralocorticoid receptor activation and cardiac fibrosis. Clin Sci 2007;112(9):467-75.
24. Arora RB, Somani P. Ectopic arrhythmia provoking action of aldosterone. Life Sci 1962;1:215–218.
25. Oberleithner H. Aldosterone makes human endothelium stiff and vulnerable. Kidney Int 2005;67:1680–1682
26. Farquharson CA, Struthers AD. Aldosterone induces acute endothelial dysfunction in vivo in humans: evidence for an aldosterone-induced vasculopathy. Clin Sci (Lond). 2002;103:425– 431.
27. Blacher J, Amah G, Girerd X, Kheder A, Ben Mais H, London GM, Safar ME. Association between increased plasma levels of aldosterone and decreased systemic arterial compliance in subjects with essential hypertension. Am J Hypertens. 1997;10:1326 –1334.
28. Struthers AD. Aldosterone: cardiovascular assault. Am Heart J. 2002; 144:S2–S7.
29. Zannad F, Radauceanu A. Effect of MR blockade on collagen formation and cardiovascular disease with a specific emphasis on heart failure. Heart Fail Rev. 2005;10:71–78.
30. Hombach V, Grebe O, Merkle N, Waldenmaier S, Hoher M, Kochs M, Wohrle J, Kestler HA. Sequelae of acute myocardial infarction regarding cardiac structure and function and their prognostic significance as assessed by magnetic resonance imaging. Eur Heart J 2005;26(6):549-57.
31. Weisman HF, Bush DE, Mannisi JA, Weisfeldt JM, Healy B. Cellular mechanisms of myocardial infarct expansion. Circulation 1988;78:186-201.
32. Whittaker P, Boughner DR, Kloner RA. Role of collagen in acute myocardial infarct expansion. Circulation 1991;84(5):2123-34.
33. Codreanu A, Odille F, Aliot E, Marie PY, Magnin-Poull I, Andronache M, Mandry D, Djaballah W, Régent D, Felblinger J, de Chillou C. Electroanatomic characterization of post-infarct scars comparison with 3-dimensional myocardial scar reconstruction based on magnetic resonance imaging. J Am Coll Cardiol. 2008;52(10):839-42.
34. Codreanu A, Djaballah W, Angioi M, Ethevenot G, Moulin F, Felblinger J, Sadoul N, Karcher G, Aliot E, Marie PY. Detection of myocarditis by contrast-enhanced MRI in patients presenting with acute coronary syndrome but no coronary stenosis. J Magn Reson Imaging. 2007;25(5):957-64.
35. Maskali F, Ayalew A, Marie PY, Menu P, Antunes L, Mertes PM, Zannad F, Gravier JM, Karcher G, Bertrand A. Changes in first-pass interstitial kinetics of DTPA in myocardium submitted to low-flow ischemia. Invest Radiol. 2005;40(12):766-72.
36. Mandry D, LAPICQUE F, Djaballah W, ESCANYE JM, ODILLE F, FELBLINGER J, Karcher G, Marie PY. Multicompartmental analysis of late contrast enhancement in areas of myocardial infarction supplied by chronically occluded coronary arteries. J Magn Reson Imaging. 2008 (sous presse).
37. Bolognese L, Neskovic AN, Parodi G, Cerisano G, Buonamici P, Santoro GM, Antoniucci D. Left ventricular remodeling after primary coronary angioplasty: patterns of left ventricular dilation and long-term prognostic implications. Circulation. 2002;106(18):2351-7.
38. Bellenger NG, Davies LC, Francis JM, Coats AJ, Pennell DJ. Reduction in sample size for studies of remodeling in heart failure by the use of cardiovascular magnetic resonance. J Cardiovasc Magn Reson 2000;2(4):271-8.
39. Bottini PB, Carr AA, Prisant LM, Flickinger FW, Allison JD, Gottdiener JS. Magnetic resonance imaging compared to echocardiography to assess left ventricular mass in the hypertensive patient. Am J Hypertens. 1995;8(3):221-8.
40. Hundley WG, Li HF, Hillis LD, Meshack BM, Lange RA, Willard JE, Landau C, Peshock RM. Quantitation of cardiac-output with velocity-encoded, phase-difference magnetic-resonance-imaging. Am J Cardiol. 1995;75(17):1250-5.
41. Mandry D, Kearney-Schwartz A, Joly L, Djaballah W, Böhme P, Escanyé JM, Vuissoz PA, Adamopoulos C, Zannad F, Marie PY. Magnetic resonance imaging characterization of the initial stage of obesity-related cardiac remodelling: Relationships with aortic stiffness and visceral fat accumulation. Etude R2C2 [abstract]. J Hypertens 2008;26(supp 1):S47.
42. Ou P, Celermajer DS, Jolivet O, Buyens F, Herment A, Sidi D, Bonnet D, Mousseaux E. Increased central aortic stiffness and left ventricular mass in normotensive young subjects after successful coarctation repair. Am Heart J. 2008; 155:187-93.
43. van der Meer RW, Diamant M, Westenberg JJ, Doornbos J, Bax JJ, de Roos A, Lamb HJ. Magnetic resonance assessment of aortic pulse wave velocity, aortic distensibility, and cardiac function in uncomplicated type 2 diabetes mellitus. J Cardiovasc Magn Reson. 2007; 9:645-51.
44. Vulliémoz S, Stergiopulos N, Meuli R. Estimation of local aortic elastic properties with MRI. Magn Reson Med. 2002; 47:649-54.
45. Safar ME, Czernichow S, Blacher J. Obesity, arterial stiffness, and cardiovascular risk. J Am Soc Nephrol. 2006; 17: S109–11.
46. Nichols WW. Clinical measurement of arterial stiffness obtained from noninvasive pressure waveforms. Am J Hypertens. 2005; 18:3S-10S.
47. Woodman RJ, Watts GF. Measurement and application of arterial stiffness in clinical research: focus on new methodologies and diabetes mellitus. Med Sci Monit. 2003; 9:RA81-9.
48. Cruickshank K, Riste L, Anderson SG, Wright JS, Dunn G, Gosling R. Aortic pulse-wave velocity and its relationship to mortality in diabetes and glucose intolerance: An integrated index of vascular function? Circulation. 2002; 106: 2085–90.
49. Host NB, Jensen LT, Bendixen PM. The aminoterminal propeptide of type III procollagen provides new information on prognosis after acute myocardial infarction. Am J Cardiol. 1995;76:869–873.
50. Radovan J, Vaclav P, Petr W, Jan C, Michal A, Richard P, Martina P. Changes of collagen metabolism predict the left ventricular remodeling after myocardial infarction. Mol Cell Biochem. 2006;293(1-2):71-8.
51. Cerisano G, Parodi G, Dovellini EV, Migliorini A, Tommasi M, Raspanti S, Buonamici P, Taddeucci E, Valenti R, Antoniucci D. Time course of serum collagen types I and III metabolism products after reperfused acute myocardial infarction in patients with and without systemic hypertension. J Hum Hypertens. 2008 (sous presse).
52. Bonapace S, Rossi A, Cicoira M, Golia G, Zanolla L, Franceschini L, Conte L, Marino P, Zardini P, Vassanelli C. Aortic stiffness correlates with an increased extracellular matrix turnover in patients with dilated cardiomyopathy. Am Heart J 2006;152(1):93.e1-6.
53. Cerisano G, Valenti R, Sciagrà R, Pucci PD, Tommasi M, Raspanti S, Pupi A, Dovellini EV, Antoniucci D. Relationship of sustained brain natriuretic peptide release after reperfused acute myocardial infarction with gated SPECT infarct measurements and its connection with collagen turnover and left ventricular remodeling. J Nucl Cardiol. 2008;15(5):644-54.
54. Ronco C, Haapio M, House AA, Anavekar N, Bellomo R. Cardiorenal syndrome. J Am Coll Cardiol. 2008;52(19):1527-39.
55. Marie PY, Djaballah W, Franken PR, Vanhove C, Muller MA, Boutley H, Poussier S, Olivier P, Karcher G, Bertrand A. OSEM reconstruction, associated with temporal fourier and depth-dependant resolution recovery filtering, enhances results from sestamibi and 201Tl 16-interval gated SPECT. J Nucl Med. 2005;46(11):1789-95.
56. Manjunath G, Sarnak MJ, Levey AS. Estimating the glomerular filtration rate. Dos and don'ts for assessing kidney function. Postgrad Med. 2001;110(6):55-62.

## Calendrier des visites

|  | **Inclusion** | **Bilan initial**  **(2 à 4 jours post STEMI)** | **Bilan à 6 mois**  **(+/- 7 jours)** |
| --- | --- | --- | --- |
| Vérification des critères d’inclusion/non inclusion | × | × |  |
| Recueil du consentement éclairé | × |  |  |
| Evaluation clinique/interrogatoire médical | × |  |  |
| Recueil des antécédents et facteurs de risque cardio-vasculaires | × |  |  |
| Recueil des modalités de traitement de reperfusion en phase aiguë | × |  |  |
| Recueil des traitements médicaux | × | × | × |
| Mesures de la pression artérielle systémique | × | × | × |
| Prélèvements sanguins pour la mesure des biomarqueurs (30ml) |  | × | × |
| IRM cardio-vasculaire |  | × | × |
| Recueil des Evènements Indésirables |  | × | × |
| Recueil des données dans le cahier d’observation | × | × | × |

## Données recueillies

Les principales données qui seront recueillies dans le cahier d’observation sont les suivantes :

- pour les données démographiques, anthropométriques et anamnestiques,

- l’âge, le poids, la taille, la surface corporelle, l’index de masse corporelle et le sexe,
- les antécédents médicaux (et en particulier les antécédents cardiovasculaires),
- les facteurs de risque cardiovasculaires,
- les modalités de traitement de reperfusion en phase aiguë (délai depuis le début des douleurs ; angioplasties uniques ou multiples, avec ou sans stenting, stent pharmacologiquement actif ou non actif), les résultats immédiats (scores TIMI, « TIMI frame count », score de Blush) et nombre d’atteintes tronculaires résiduelles,
- les traitements médicamenteux et en particulier ceux ayant un effet cardio-protecteur possible ou certain (IEC, anti-aldostérones, bêta-bloquants, statines…),
- résultats d’examens biologiques récents (créatininémie, urée sanguine, ionogramme sanguin…),

- pour les paramètres recueillis et/ou mesurés lors des bilans initiaux et à six mois,

- les paramètres cardiaques, en particulier ceux témoignant de la mesure de la taille de l’infarctus (analyses quantitative et semi-quantitative visuelle), mais aussi les volumes télédiastolique et télésystolique du ventricule gauche, la fraction d’éjection du ventricule gauche, la masse myocardique ventriculaire gauche, le débit cardiaque et l’index cardiaque,
- les paramètres mesurés en IRM et témoignant des conditions de charge (résistances périphériques totales, vélocité de l’onde de pouls aortique),
- les valeurs de la pression artérielle systémique mesurées lors de l’IRM (valeurs médianes des pressions systolique, diastolique et moyenne),
- l’aldostéronémie et l’ensemble des autres biomarqueurs mesurés dans le cadre de l’étude (CRP-US, BNP, rénine, créatininémie à 6 mois),
- les évènements indésirables et complications depuis l’infarctus,
- les modifications du traitement médical depuis l’infarctus.

## Mesures prises pour éviter les biais

Tous les paramètres d’IRM cardiovasculaire seront mesurés par les 2 mêmes observateurs expérimentés. Une deuxième analyse consensuelle sera systématiquement réalisée lorsque ces mesures seront considérées comme étant éloignées et ce sont alors les nouvelles valeurs consensuelles qui seront utilisées dans les analyses ultérieures. Dans le cas contraire, ce sont les moyennes des 2 mesures qui seront utilisées.

## Durée de l’étude

**Durée de participation pour un patient :** 6 mois

**Durée théorique de recrutement pour atteindre le nombre de sujets nécessaires** : 18 mois

**Date théorique de début d’étude :** dernier trimestre 2009

**Date théorique de fin d’étude** : dernier trimestre 2011

## Gestion des arrêts prématurés et des exclusions en cours d’étude

### Gestion des arrêts prématurés

Les patients susceptibles d’interrompre prématurément l’étude sont les patients perdus de vue, les patients dans l’impossibilité de passer le bilan avec l’IRM à 6 mois (état instable, apparition d’une ACFA, implantation d’un pace maker ou d’un défibrillateur…) ou ayant retiré leur consentement. Ces patients ne seront pas remplacés, l’effectif requis par le protocole prévoyant 20 % de patients non évaluables à 6 mois (refus ou impossibilité de passer le bilan à 6 mois), ce pourcentage étant celui observé dans 2 études réalisées en IRM dans des conditions similaires (5, 30).

Etant donnée la nature de l’étude, aucune mesure particulière de prise en charge ou de suivi médical des sujets ne sera mise en place à la fin de la participation de chaque patient ni en cas d’arrêt prématuré de l’étude.

### Exclusions en cours d’étude

Aucune situation ne conduira à l’exclusion d’un patient de l’étude.

### Arrêt de l’étude

Le promoteur se réserve le droit d'interrompre l'essai à tout moment pour des raisons médicales ou administratives. Dans cette éventualité, une lettre d’information sera adressée aux investigateurs associés. Les patients inclus devront interrompre l’étude mais continueront à être suivis par l’investigateur en dehors du protocole. L’AFSSAPS ainsi que le CPP Est III ayant statué sur le protocole seront également informés par courrier par le Promoteur.

## Participation simultanée à une autre recherche

Les volontaires pourront participer simultanément à une autre recherche pendant la durée de l’étude. Il n’y a pas de période d’exclusion prévue à l’issue de la recherche.

# Sélection et exclusion des personnes de la recherche :

## Critères d’inclusion

- Homme ou femme hospitalisé(e) dans le service de cardiologie du CHU de Nancy pour un primo-infarctus myocardique avec sus décalage du segment ST revascularisé en phase aiguë par angioplastie primaire et datant de moins de 4 jours. L’infarctus myocardique aigu avec sus-décalage du segment ST (STEMI) sera défini par la présence des critères suivants : douleur thoracique prolongée, augmentation des enzymes cardiaques (CPK-MB > 2 fois la limite supérieure de la normale), et présence d’un sus décalage du segment ST d’au moins 2 mm et touchant au moins deux dérivations contiguës de l’ECG,
- Patient présentant un état clinique stable (sans signe d’insuffisance cardiaque ou coronaire instable, sans HTA mal stabilisée sous traitement),
- Patient présentant un rythme cardiaque sinusal régulier
- Patient ayant un âge ≥ 18 ans
- Patient ayant reçu la lettre d’information spécifique à l’étude et ayant signé le formulaire de consentement éclairé

## Critères de non inclusion

- Contre-indication à l’examen IRM (pacemaker, clip métallique neurochirurgical…)
- Claustrophobie sévère
- Antécédent d’hypersensibilité aux sels de gadolinium
- Cardiopathie d’origine non ischémique surajoutée (cardiopathie valvulaire avec rétrécissement serré ou fuite de grade > 2, cardiopathie congénitale, cardiopathie hypertrophique, cardiopathie dilatée préexistante connue)
- Nécessaire programmation d’une chirurgie cardiaque dans les 6 mois (pontage aorto-coronaire, chirurgie valvulaire …)
- Femme en âge et en état de procréer et dépourvue de méthode contraceptive efficace
- Absence de couverture sociale
- Patient incapable de se plier au suivi de l’étude
- Patient majeur sous tutelle, sous curatelle ou sous sauvegarde de justice

# Traitement administré aux personnes qui se prêtent à la recherche

Pour la réalisation de l’IRM cardiovasculaire, le produit de contraste utilisé sera le DOTAREM^TM^ (Laboratoire GUEBERT, France). Le RCP de ce produit se trouve en annexe (cf annexe Error: Reference source not found ).

# Evaluation de l’efficacité

Il n’y a pas d’évaluation de l’efficacité dans ce protocole.

# Evaluation de la sécurité

## Description des paramètres d’évaluation de la sécurité

Les documents de référence sont les RCP du produit de contraste utilisé : DOTAREM^TM^ (Laboratoire GUEBERT, France, cf annexe Error: Reference source not found).

## Méthodes et calendrier prévus pour mesurer, recueillir et analyser les paramètres d’évaluation de la sécurité

Le recueil des informations sur la sécurité du patient sera effectué de manière systématique lors de chacune des visites prévues par le protocole.

## Procédures mises en place en vue de l’enregistrement et de la notification des évènements indésirables

### Définitions

Un **événement indésirable** est une manifestation nocive survenant chez une personne qui se prête à une recherche biomédicale, que cette manifestation soit liée ou non à la recherche ou au produit sur lequel porte cette recherche.

Un **effet indésirable** est une réaction nocive et non voulue liée à un médicament expérimental, se produisant à la posologie normalement utilisée chez l’homme

**Un évènement indésirable grave ou effet indésirable grave** **(EIG)** est un évènement ou un effet ayant pu contribuer au décès du patient, à la mise en jeu de son pronostic vital, à son hospitalisation ou à la prolongation de son hospitalisation, à une incapacité ou à un handicap important ou durable, ou se traduire par une anomalie ou une malformation congénitale

**La liste des effets indésirables attendus** liés à l’étude est établie à partir du document de référence.

Un EIG est inattendu (EIGI) lorsqu’il ne figure pas sur cette liste.

**Un fait nouveau** peut être : fréquence inattendue d’un EIG attendu, événement indésirable grave lié à la procédure de l’essai, efficacité insuffisante dans les maladies à pronostic vital, données non cliniques.

### Document de référence permettant de définir le caractère attendu d’un EIG

Pour la réalisation de l’IRM cardiovasculaire, le produit de contraste utilisé sera le DOTAREM^TM^ (Laboratoire GUEBERT, France). Le RCP de ce produit se trouve en annexe (cf annexe Error: Reference source not found).

### Liste des effets indésirables attendus

Voir RCP en annexe Error: Reference source not found.

### Transmission des EIG et faits nouveaux

Dès qu’un investigateur prend connaissance d’un EIG ou d’un fait nouveau, il le déclare sans délai au promoteur en faxant la fiche de déclaration d’EIG (voir annexe Error: Reference source not found) au **03 83 85 98 14**.

- S’il s’agit d’un effet indésirable grave inattendu (EIGI) ou si c’est un fait nouveau, le promoteur entre en contact avec l’investigateur pour rédiger un rapport initial qui sera transmis à l’AFSSAPS et au CPP dans les 7 jours. La transmission à l’AFSSAPS se fera par fax au n° 01 55 87 36 63 ou par courrier avec accusé de réception.

Lorsque l’évènement n’est pas résolu à la date d’envoi du fax, l’investigateur est tenu d’envoyer un rapport complémentaire afin de documenter l’évolution ou de réactualiser les données manquantes.

- S’il s’agit d’un effet indésirable grave attendu, il sera colligé en vue de la rédaction des rapports annuels de sécurité.

### Transmission des effets indésirables non graves

Ils seront décrits succinctement par l’investigateur sur la fiche récapitulative dédiée à cet effet dans le cahier d’observation. Les informations recueillies seront : nature de l’évènement, date d’apparition ou de modification d’intensité, intensité, relation avec l’objet de l’étude, les mesures prises, l’évolution en fin d’étude.

## Modalités et durée de suivi des personnes suite à la survenue d’évènements indésirables

Lorsque un évènement indésirable grave persiste, y compris après la fin de l’étude, l’investigateur suivra le patient jusqu’à ce que l’évènement soit considéré comme résolu et transmettra les données de suivi au promoteur**.**

## Comité de surveillance indépendant

Etant donné la nature de cette étude, il n’y a pas de justification à constituer un comité de surveillance indépendant

## Rapports de sécurité

- Déclaration semestrielle : le promoteur rédige la liste des suspicions d’EIG inattendus survenus dans une autre recherche qu’il conduit en France ou survenus hors du territoire français dans l’essai concerné pendant la période considérée, ainsi qu’une synthèse concise mettant en exergue les principaux problèmes de sécurité soulevés.
- Rapports annuels de sécurité : le promoteur rédige les rapports annuels de sécurité et les transmet à l’AFSSAPS, au CPP et à l’investigateur coordonnateur. L’investigateur coordonnateur transmettra au promoteur toutes les données nécessaires à la rédaction de ce rapport.

# Statistiques

## Considérations générales

Toutes les analyses seront effectuées à l'aide du logiciel SAS® V9.1.3 (SAS Institute, Cary, NC, USA). Les analyses inférentielles seront effectuées en situation bilatérale avec un seuil de signification de 5 %. Les données manquantes ne seront pas reconstituées.

**Une analyse rétrospective** sur 2 ans (d’octobre 2006 à octobre 2008) a permis d’estimer qu’environ 230 patients présentaient chaque année les critères de sélection de notre étude, ce qui devrait permettre d’inclure la totalité de la population de l’étude (250 patients) en 18 mois.

Pour le calcul de l’effectif, nous nous sommes basés sur les **résultats d’une étude très récente**, elle aussi réalisée chez des patients revascularisés en phase aiguë d’un STEMI et où une IRM fut effectuée à J2-J4 puis à 4 mois (5). Dans cette étude, l’IRM a mis en évidence un remodelage délétère chez 17 % des patients. Celui-ci était défini de manière conventionnelle, par une augmentation de 20 % du volume télédiastolique, et il était alors associé à une forte détérioration du pronostic, confirmant ainsi les résultats de précédentes études (30, 37).

La durée de suivi en IRM étant plus importante dans notre étude (6 mois), nous pouvons donc compter sur une fréquence plus importante de remodelage, au moins 20 %. On peut d’ailleurs noter que les études échographiques, qui ont été réalisées dans le même contexte, ont permis d’observer des pourcentages de remodelage proche de 30 % à ce délai de 6 mois (37), même si l’échographie s’avère très probablement moins précise que l’IRM pour détecter des variations du volume ventriculaire gauche (38).

Dans cette étude, il sera indispensable de pouvoir non seulement rechercher une relation entre aldostéronémie et remodelage délétère, mais aussi de démontrer **l’indépendance de cette relation vis-à-vis des principaux déterminants du remodelage cardiaque**. Cette indépendance sera recherchée dans des analyses multivariées dont les possibilités dépendent de la fréquence de l’évènement recherché, qui est ici la survenue d’un remodelage délétère à 6 mois. Nous avons identifié 3 principaux déterminants du remodelage, qu’il faudra pourvoir analyser conjointement à l’aldostéronémie dans ces analyses multivariées, **ce qui nécessitera la présence d’au moins 40 patients avec remodelages délétère à 6 mois** (10 par variable testée). Ces 3 principaux déterminants sont la taille initiale de l’infarctus mesurée en IRM (2-7)) et deux paramètres cliniques : l’âge (2, 8) et l’hypertension artérielle (2, 10). D’autres paramètres, qui seront pourtant eux aussi recueillis et analysés, n’ont pas été retenus parmi ces déterminants essentiels. Il s’agit en particulier de la prescription d’un inhibiteur de l’enzyme de conversion de l’angiotensine (IEC (2)), qui a un effet bénéfique car antagoniste du remodelage délétère. Cependant les IEC ne sont plus a priori un facteur discriminant du remodelage délétère puisqu’ils sont actuellement prescrits chez la très grande majorité des patients victimes d’un infarctus myocardique.

D’autres n’ont été qu’inconstamment retrouvés comme étant des paramètres prédictifs indépendants du remodelage. Il s’agit en particulier du diabète, de l’infarctus de localisation antérieure, de la présence d’une atteinte multi-tronculaire, de la présence d’une atteinte restrictive initiale, et de la prescription de certains autres médicaments (bêta-bloquants, statines). Cependant, les analyses multivariées seront adaptées aux résultats des analyses univariées et certains de ces paramètres pourront donc être ajoutés ou soustraits à la liste prévisionnelle des 4 variables testées.

### Justificatif de l’effectif

Avec un pourcentage de remodelage délétère estimé à 20 % et la nécessité d’obtenir au moins 40 patients avec remodelage délétère, un nombre minimal de 200 patients devrait être inclus dans cette étude. Au final, **il serait souhaitable d’inclure près de 250 patients,** si on considère que plus de 20 % pourraient refuser ou ne pas pouvoir réaliser l’examen de contrôle à 6 mois (état instable, apparition d’une ACFA, implantation d’un pace-maker ou d’un défibrillateur). Rappelons que ce pourcentage de 20 % est celui observé dans 2 études réalisées en IRM dans des conditions similaires (5, 30).

Ainsi, avec un effectif de 200 patients dont 40 présentant un modelage délétère à 6 mois, l'essai aura une puissance de 80 % de mettre en évidence avec un risque d'erreur bilatéral de 5 % une différence supérieure ou égale à 0.54 déviation standard pour les comparaisons inter-groupes (valeurs initiales et évolution sur 6 mois) et un coefficient de corrélation r supérieur ou égal à 0.20 entre 2 paramètres quelconques.

### Variables quantitatives continues

Les variables continues seront décrites en effectif, moyenne et écart type. Leurs distributions seront étudiées par des tests de Shapiro-Wilk, complétés par l'examen des coefficients standardisés de dissymétrie (skewness) et de courbure (kurtosis). En cas de défaut de normalité, les analyses utiliseront les tests non-paramétriques s'il en existe (Wilcoxon sur séries appariées, corrélation de Spearman, …) ou rechercheront une transformation préalable standardisant les variables concernées (logarithmique, ...) s’il n’en existe pas.

### Variables qualitatives discontinues

Les variables discontinues seront présentées en effectifs et pourcentages.

## Analyses réalisées pour répondre à l’objectif principal

Rappel de l’objectif principal : déterminer si l’aldostéronémie s’avère prédictive de l’apparition d’un remodelage dans les 6 mois suivant un STEMI revascularisé en phase aiguë, indépendamment des paramètres prédictifs conventionnels (taille de l’infarctus, âge, HTA).

**Les variations du volume télédiastolique ventriculaire gauche** seront déterminées entre l’examen initial et le contrôle à 6 mois. Les patients, ayant une augmentation de plus de 20 % du volume initial et dont on connaît la signification pronostique péjorative (5, 30, 37), seront identifiés. Rappelons qu’ils devraient constituer au moins 20 % de la population suivie à 6 mois et donc, environ 40 patients.

**Les analyses univariées** permettront d’identifier les paramètres prédictifs du remodelage (patients avec augmentation de volume > 20 %, différence entre volume à 6 mois et volume initial) parmi :

1) les principales variables cliniques d’intérêt (l’âge ; les facteurs de risque cardio-vasculaires et en particulier l’HTA ; les antécédents médicaux et en particulier les antécédents cardiovasculaires) ;

2) les modalités de traitement de reperfusion en phase aiguë (délai depuis le début des douleurs ; angioplasties uniques ou multiples, avec ou sans stenting, stent pharmacologiquement actif ou non actif), les résultats immédiats (scores TIMI, « TIMI frame count », score de Blush) et nombre d’atteintes tronculaires résiduelles,

3) les traitements prescrits à la sortie de l’hôpital et lors du contrôle à 6 mois, en particulier ceux ayant un effet cardioprotecteur possible ou certain (IEC, anti-aldostérones, bêta-bloquants, statines, …),

4) l’aldostéronémie, mesurée lors de l’examen initial et à 6 mois, qui constitue le sujet d’étude essentiel, mais aussi l’ensemble des autres paramètres biologiques mesurés (CRP-US, BNP, rénine, rapport aldostérone / rénine, créatininémie permettant la mesure de la clairance de la créatinine selon la formule MDRD),

5) les paramètres cardiaques du bilan initial, en particulier ceux témoignant de la mesure de la taille de l’infarctus (analyses quantitative et semi-quantitative visuelles), mais aussi les volumes télédiastolique et télésystolique du ventricule gauche, la fraction d’éjection du ventricule gauche, le masse myocardique ventriculaire gauche, le débit cardiaque et l’index cardiaque,

6) les paramètres témoignant des conditions de charge et qui seront mesurés lors du bilan initial et du bilan à 6 mois effectué en IRM (pressions artérielles systolique, diastolique et moyenne ; résistances périphériques totales, vélocité de l’onde pouls aortique).

**Des analyses multivariées** seront réalisées pour rechercher les principaux paramètres prédictifs indépendants de l’importance du remodelage (différence entre volumes télédiastoliques mesurés à 6 mois et lors du bilan initial) et de la présence d’un remodelage délétère (augmentation de ce volume de plus de 20 %), parmi ceux présentant une valeur de P < 0,1 en analyse univariée. En particulier, si l’aldostéronémie s’avère être un paramètre prédictif en analyse univariée, il sera déterminé si cette prédiction est indépendante et additive à celle des autres paramètres sélectionnés.

## Analyses réalisées pour répondre aux objectifs secondaires

Rappel des objectifs secondaires : individualiser des mécanismes potentiels et/ou des situations propices à l’action délétère de l’aldostérone sur le remodelage en recherchant des associations entre d’une part, l’aldostéronémie et d’autre part, les paramètres témoignant : (i) du fonctionnement vasculaire, (ii) du fonctionnement cardiaque, (iii) des signes biologiques de fibrose, d’inflammation systémique, de tension pariétale excessive et de dysfonction rénale et (iii) de certaines situations cliniques (HTA, infarctus étendu, âge avancé…).

- Pour la recherche de mécanismes, des corrélations seront recherchées entre, d’une part, les mesures d’aldostéronémie lors du bilan initial et à 6 mois et, d’autre part, les valeurs initiales et les variations à 6 mois de paramètres susceptibles d’interagir avec le remodelage et pour lesquels on peut suspecter une influence de l’aldostérone. Il s’agit en particulier de paramètres témoignant : (i) du fonctionnement vasculaire (pression artérielle, rigidité aortique et résistances périphériques mesurées en IRM), (ii) du fonctionnement cardiaque (débit et index cardiaque, volumes télésystolique et télédiastolique du ventricule gauche, fraction d’éjection du ventricule gauche, étendue de l’infarctus) et (iii) de l’activité de fibrose, de l’inflammation systémique et de la tension pariétale (concentrations plasmatiques de PIIINP, CRP ultra sensible, BNP).
- Pour la recherche de situations propices à l’action délétère de l’aldostérone, nous rechercherons des corrélations entre, d’une part l’aldostéronémie, et d’autre part certains paramètres susceptibles d’induire une majoration des concentrations sériques en aldostérone, en particulier l’âge, l’antécédent d’HTA, l’étendue des séquelles d’infarctus, les paramètres permettant de quantifier la sévérité de la dysfonction cardiaque initiale (fraction d’éjection, volumes télédiastolique et télésystolique), la réninémie et des signes biologiques de dysfonction rénale (créatininémie et clairance de la créatinine selon la formule MDRD).

# Droit d'accès aux données et documents sourceS.

Toutes les données et informations concernant le patient resteront strictement confidentielles.

Les données concernant celui-ci ne seront accessibles qu’aux personnes participant à cette recherche et aux personnes chargées par le promoteur de contrôler la qualité de l’étude ; le cas échéant elles pourront également être transmises aux autorités sanitaires habilitées. Dans tous les cas, elles seront exploitées dans les conditions garantissant leur confidentialité.

# Contrôle et assurance de la qualité.

Le contrôle qualité sera effectué par les Attachées de Recherche Clinique de la Direction de la Recherche et de l’Innovation (DRI), mandatées par le promoteur.

La nature et la fréquence du monitoring seront établies selon la grille monitoring/risque établie.

L’ARC de la DRI contrôlera, tout au long de l’étude, lors de visites de monitoring planifiées avec l’investigateur :

⮚ Les données recueillies au cours de l’étude

⮚ Les consentements de tous les patients inclus

A ce titre, l’investigateur s’engage à mettre à la disposition de l’ARC lors de ses visites de monitoring :

⮚ Les dossiers médicaux des patients

⮚ Les cahiers de recueil de données

⮚ Les formulaires de consentement des patients inclus

Ce monitoring effectué par les ARC de la DRI permettra d’évaluer :

⮚ La protection des personnes

⮚ La fiabilité des données par rapport aux documents sources

⮚ La conformité de l’essai par rapport au protocole, aux Bonnes Pratiques Cliniques et à la législation en vigueur en matière de recherches biomédicales

Au terme de ce contrôle qualité, un **rapport de monitoring** sera rédigé par l’ARC et remis au Directeur chargé de la Recherche qui prendra des directives en fonctions des conclusions de ce rapport.

# Considérations éthiques

La recherche sera conduite conformément au protocole, aux bonnes pratiques en vigueur et aux dispositions législatives et réglementaires en vigueur.

## FICHIER NATIONAL

Les patients de cette étude ne seront pas recensés dans le fichier national des volontaires se prêtant à une recherche biomédicale.

## CPP/AFSSAPS

Le protocole et le formulaire d'information et de consentement de l'étude seront soumis pour analyse et avis au CPP Est III et à l’AFSSAPS.

Les notifications des avis du CPP et de l’AFSSAPS seront fournies au promoteur de l'étude qui en transmettra une copie à chacun des investigateurs avant le début de l'étude.

## AMENDEMENT (= modification substantielle)

Si des amendements au protocole, c'est-à-dire qui en modifient le sens ou les objectifs ou qui modifient les contraintes subies ou les risques encourus par les participants, s'avèrent nécessaires ils seront d'abord soumis à l'avis du promoteur de l'étude. Après réception de l'accord du promoteur, ces amendements seront ensuite soumis à l'avis du CPP et de l’autorité compétente (AFSSAPS). Les modifications prévues dans l’amendement au protocole ne pourront être mises en œuvre qu’après la réception de l’avis favorable de ces 2 instances.

## CONSENTEMENT

Avant de recueillir le consentement du patient, l’investigateur s’engage lui à délivrer une information claire et la plus complète possible sur l'étude envisagée ; il lui remet également la notice d’information.

L’investigateur s’engage à recueillir le consentement éclairé de chaque patient avant toute investigation ou consultation induite de façon spécifique par la recherche biomédicale

Le consentement écrit et signé doit IMPERATIVEMENT être recueilli AVANT toute participation du patient à l'étude, en particulier avant toute randomisation

Le formulaire de consentement sera signé en trois exemplaires par le sujet et le médecin investigateur :

⮚ Un exemplaire sera remis à la personne participant à la recherche

⮚ Un exemplaire sera conservé et archivé par l'investigateur

⮚ Un exemplaire sera récupéré par l’ARC lors des visites de monitoring et archivé par le promoteur

## Assurance

Le promoteur souscrira pour toute la durée de l'étude une assurance garantissant sa propre responsabilité civile ainsi que celle de tout intervenant impliqué dans la réalisation de l'étude, indépendamment de la nature des liens existant entre les intervenants et le promoteur.

## Résultats globaux

Les résultats globaux de la recherche seront communiqués à tous les patients par le biais d’un courrier envoyé par l’investigateur coordonnateur.

# Traitement des données et conservation des documents et des données relatives à la recherche

## Protection des données

L’étude nécessitant un traitement automatisé de données de santé, une déclaration préalable sera effectuée au Comité Consultatif sur le Traitement de l'Information en Matière de Recherche dans le domaine de la Santé (CCTIMRS) puis à la Commission Nationale Informatique et Liberté (CNIL). Le traitement sera autorisé après la publication d’un acte réglementaire par le Directeur du CHU.

En application de la loi « informatique, fichiers et liberté » du 06 janvier 1978 modifiée, le fichier informatique utilisé pour réaliser la présente recherche fera l’objet d’une autorisation de la CNIL. Seules les données nécessaires à la recherche seront recueillies. Le patient a néanmoins le droit de s’opposer à ce que les données le concernant fassent l’objet d’un traitement automatisé. Le patient aura à tout moment le droit d’accéder aux données le concernant. Il aura également le droit de demander à ce que les données inexactes ou devenues inexactes soient rectifiées. Il pourra à tout moment exercer ces droits auprès du Dr Angioi, investigateur coordonnateur.

Pour toutes les informations de nature médicale, ces droits pourront être exercés directement ou par l’intermédiaire du médecin de son choix.

## Archivage des documents

L’archivage des documents réglementaires se fera pendant au moins 30 ans.

# Financement

Cette étude est soumise à l’appel à projets interrégional du Programme Hospitalier de Recherche Clinique (PHRC) 2009.

# Règles relatives à la publication.

Les premier et dernier auteurs des publications qui seront issues de cette étude seront des praticiens hospitaliers du CHU de Nancy.

En plus de l’affiliation à son service d’origine, l’investigateur coordonnateur devra également être cité comme affilié au CIC de Nancy selon le modèle suivant :

CIC INSERM-CHU de Nancy, Centre d’Investigation Clinique de Nancy CIC 9501, Dommartin-lès-Toul, F-54201, France.

# Liste des annexes

## Annexe 1 liste des investigateurs

**Investigateur coordonnateur :**

Michael ANGIOI

Service de Cardiologie

Hôpital de Brabois

Téléphone : 03 83 15 32 40

Télécopie : 03 83 15 42 13

m.angioi@chu-nancy.fr

**Investigateurs associés :**

Pierre-Yves MARIE, Médecine Nucléaire, CHU de Nancy,

Wassila DJABALLAH, Médecine Nucléaire, CHU de Nancy,

Damien MANDRY, Radiologie, CHU de Nancy,

Gilles BOSSER, Institut de Réadaptation Fonctionnelle de Nancy,

Andrei CODREANU, Service de Cardiologie, CH du Luxembourg,

Patrick LACOLLEY, INSERM U684 Faculté de Médecine de Nancy,

Athanase BENETOS, Gériatrie, CHU de Nancy,

Jacques FELBLINGER, CIC-IT , CHU de Nancy,

Cédric PASQUIER, CIC-IT, CHU de Nancy,

Faiez ZANNAD, CIC-P, CHU de Nancy,

Patrick ROSSIGNOL, CIC-P, CHU de Nancy

## Annexe 2 RCP DOTAREM ^TM^

RÉSUMÉ DES CARACTÉRISTIQUES DU PRODUIT

Mis à jour : 31/05/2007

1. DENOMINATION DU MEDICAMENT

DOTAREM 0,5 mmol/ml, solution injectable

2. COMPOSITION QUALITATIVE ET QUANTITATIVE

Acide gadotérique^*^ .......................................................................................................................... 27,932 g

Correspondant à DOTA ................................................................................................................... 20,246 g

Correspondant à oxyde de gadolinium ................................................................................................ 9,062 g

Pour 100 mL de solution.

^*^Acide gadotérique: complexe de gadolinium de l'acide 1, 4, 7, 10 tétra-azacyclododécane N, N', N'', N''' tétra-acétique.

Concentration en produit de contraste: 0,5 mmol/mL

Osmolalité: 1 350 mOsm.kg^-1^

Viscosité à 20° C: 3,2 mPa.s

Viscosité à 37° C: 2,0 mPa.s

pH: 6,5 à 8,0

Pour la liste complète des excipients, [voir rubrique 6.1](http://afssaps-prd.afssaps.fr/php/ecodex/rcp/R0121435.htm#Rcp_6_1_ListeExcipients%23Rcp_6_1_ListeExcipients).

3. FORME PHARMACEUTIQUE

Solution injectable en flacon.

4. DONNEES CLINIQUES

4.1. Indications thérapeutiques

Imagerie par résonance magnétique pour:

 pathologies cérébrales et médullaires,

 pathologies du rachis,

 et autres pathologies du corps entier (dont angiographie).

4.2. Posologie et mode d'administration

La dose recommandée est de 0,1 mmol/kg soit 0,2 mL/kg, chez l'adulte comme chez l'enfant et le nourrisson.

En angiographie, lorsque les résultats de l'examen en cours le rendent nécessaire, une deuxième injection au cours de la même session est possible.

Dans quelques cas exceptionnels comme la confirmation du caractère unique d'une métastase ou la détection de tumeurs leptoméningées, une deuxième injection de 0,2 mmol/kg peut être administrée.

Le produit doit être administré en injection intraveineuse stricte.

4.3. Contre-indications

Antécédents d'hypersensibilité aux sels de gadolinium.

Contre-indications liées à l'IRM:

 sujet porteur de pace-maker,

 sujet porteur de clip vasculaire.

4.4. Mises en garde spéciales et précautions d'emploi

A administrer uniquement par voie intraveineuse stricte. En cas d'extravasation du produit, on peut observer des réactions d'intolérance locale nécessitant des soins locaux courants.

DOTAREM ne doit pas être administré en injection subarachnoïdienne (ou épidurale).

 **Réactions de type anaphylactique:**

Comme pour les autres produits de contraste contenant du gadolinium, des réactions de type anaphylactique peuvent survenir ([voir rubrique 4.8](http://afssaps-prd.afssaps.fr/php/ecodex/rcp/R0121435.htm#Rcp_4_8_EffetsIndesirables_2%23Rcp_4_8_EffetsIndesirables_2)). La plupart de ces réactions surviennent dans un délai d'une demi-heure après l'injection du produit de contraste.

Cependant, comme avec les autres produits de contraste de cette classe, on ne peut exclure la possibilité de réactions tardives, survenant plusieurs jours après l'injection.

Compte tenu de ces risques, les patients doivent être interrogés avant toute injection sur d'éventuels antécédents allergiques (p. ex. rhume des foins, urticaire, asthme, etc.) et/ou sur une éventuelle réaction antérieure aux produits de contraste. Ces patients ont un risque accru de réaction sévère.

L'utilisation de DOTAREM chez de tels patients ne doit être décidée qu'après évaluation minutieuse du rapport bénéfice/risque.

Comme l'indique l'expérience acquise avec les produits de contraste iodés, les réactions de type anaphylactique peuvent être aggravées chez les patients recevant des bêta-bloquants, surtout en présence d'asthme bronchique. Ces patients peuvent ne pas répondre aux traitements standards des réactions de type anaphylactique à base de bêta-agonistes.

L'examen doit être effectué sous la supervision d'un médecin. En cas de réaction de type anaphylactique, l'administration du produit de contraste doit immédiatement être interrompue et - si nécessaire - un traitement spécifique doit être instauré.

Une voie d'abord veineuse doit donc être maintenue tout au long de l'examen. Afin de permettre la prise de mesures d'urgence, les médicaments appropriés (par ex. adrénaline et antihistaminiques) doivent être disponibles, ainsi que le matériel d'intubation endotrachéal et un respirateur artificiel.

 **Insuffisance rénale**

La prudence est recommandée chez les patients atteints d'insuffisance rénale sévère.

Des cas de fibrose néphrogénique systémique (FNS) ont été rapportés après injection de certains produits de contraste contenant du gadolinium chez des patients ayant une insuffisance rénale sévère (Débit de Filtration Glomérulaire < 30 mL / min/ 1,73 m^2^). Etant donné qu'il est possible que des cas de FNS surviennent avec DOTAREM, ce produit doit être utilisé avec précaution chez ces patients.

 **Troubles du système nerveux central:**

Comme avec les autres produits de contraste contenant du gadolinium, des précautions particulières doivent être prises chez les patients dont le seuil épileptogène est abaissé. Des mesures de prudence, telles qu'une surveillance rapprochée, doivent être prises. Il convient de s'assurer de la disponibilité et de la proximité de tout matériel et médicament nécessaires pour traiter les convulsions éventuelles.

4.5. Interactions avec d'autres médicaments et autres formes d'interactions

Il n'y a aucune interaction connue à ce jour.

4.6. Grossesse et allaitement

Les études chez l'animal n'ont pas montré d'effet tératogène. En l'absence d'effet tératogène chez l'animal, un effet malformatif dans l'espèce humaine n'est pas attendu. En effet, à ce jour, les substances responsables de malformations dans l'espèce humaine se sont révélées tératogènes chez l'animal au cours d'études bien conduites sur 2 espèces.

Il n'existe pas actuellement de données en nombre suffisant, pour évaluer un éventuel effet malformatif ou fœtotoxique de l'acide gadotérique lorsqu'il est administré pendant la grossesse.

En conséquence l'utilisation de DOTAREM ne doit être envisagée au cours de la grossesse que si nécessaire.

Les données expérimentales montrent que le passage de DOTAREM dans le lait maternel est très faible (< 1 %). Aucune donnée clinique n'est disponible à ce sujet.

En conséquence, il est prudent d'interrompre transitoirement l'allaitement pendant les jours qui suivent l'examen pratiqué avec DOTAREM.

4.7. Effets sur l'aptitude à conduire des véhicules et à utiliser des machines

Les effets sur l'aptitude à conduire des véhicules et à utiliser des machines n'ont pas été étudiés.

4.8. Effets indésirables

Au cours des études cliniques, des céphalées et des paresthésies ont été très fréquemment observées (> 10 %), tandis qu'une sensation de chaleur, de froid ou de douleur au site d'injection, des nausées, vomissements, réactions cutanées de type érythème et prurit ont été fréquemment observés (> 1 % - < 10 %).

Depuis la commercialisation, d'autres effets indésirables ont été rapportés:

 **Réactions de type anaphylactique**: de rares réactions de type anaphylactique ont été rapportées. Elles peuvent être exceptionnellement sévères ou même avoir une issue fatale, notamment chez les patients ayant des antécédents allergiques.
Ces réactions de type anaphylactique peuvent survenir quelle que soit la quantité administrée et peuvent se manifester sous la forme d'un ou plusieurs symptômes suivants: angiœdème, choc anaphylactique, arrêt circulatoire et cardiaque, hypotension, œdème laryngé, bronchospasme, laryngospasme, œdème pulmonaire, dyspnée, stridor, toux, prurit, rhinite, éternuements, conjonctivite, douleur abdominale, douleur thoracique, urticaire et érythème. Certains de ces symptômes peuvent être annonciateurs d'un état de choc débutant. Des réactions tardives aux produits de contraste sont possibles ([voir rubrique 4.4](http://afssaps-prd.afssaps.fr/php/ecodex/rcp/R0121435.htm#Rcp_4_4_MisesEnGarde_1%23Rcp_4_4_MisesEnGarde_1)).

 **Troubles généraux et accidents liés au site d'administration:**

o troubles généraux (très rares): malaise, sudation excessive, sensation de froid, pâleur et syncope.

o accidents liés au site d'administration: de très rares cas d'extravasation du produit ont été rapportés ([voir rubrique 4.4](http://afssaps-prd.afssaps.fr/php/ecodex/rcp/R0121435.htm#Rcp_4_4_MisesEnGarde_1%23Rcp_4_4_MisesEnGarde_1)).

 **Troubles cutanés et des tissus sous-cutanés** (très rares): eczéma, érythème.

 **Troubles du système nerveux** (très rares): convulsions généralisées.

 **Troubles musculosquelettiques, du tissus conjonctif et des os** (très rares): crampes musculaires, faiblesse musculaire.

4.9. Surdosage

Aucun cas de surdosage n'a été rapporté.

5. PROPRIETES PHARMACOLOGIQUES

5.1. Propriétés pharmacodynamiques

**PRODUIT DE CONTRASTE INTRAVASCULAIRE POUR IRM** (**V: Divers**).

L'acide gadotérique possède des propriétés paramagnétiques qui permettent le rehaussement du contraste en IRM. Il n'a pas d'activité pharmacodynamique spécifique et se révèle d'une grande inertie biologique.

5.2. Propriétés pharmacocinétiques

Injecté par voie intraveineuse, l'acide gadotérique se distribue principalement dans l'ensemble des liquides extracellulaires de l'organisme. Il n'est pas lié à l'albumine plasmatique et ne franchit pas la barrière hémato-encéphalique saine.

Chez les patients à fonction rénale normale, la demi-vie plasmatique est d'environ 90 minutes. L'élimination se fait par filtration glomérulaire sous forme inchangée.

La clairance plasmatique est ralentie en cas d'insuffisance rénale.

L'acide gadotérique est faiblement excrété dans le lait et passe lentement la barrière placentaire.

5.3. Données de sécurité préclinique

La toxicité aiguë de l'acide gadotérique injecté par voie intraveineuse a été étudiée chez la souris et le rat. Les résultats montrent que l'apparition d'effets (signes convulsifs, troubles respiratoires fugaces) survient pour des doses très éloignées de celles prévues en clinique.

L'administration d'acide gadotérique à des doses quotidiennes allant jusqu'à 15 fois la dose envisagée en clinique et pendant 28 jours, ne provoque pas d'effet notable en dehors d'une vacuolisation réversible des cellules tubulaires proximales du rein.

Aucun effet tératogène n'a été mis en évidence chez le rat et le lapin.

Aucun effet mutagène n'a été mis en évidence sur les systèmes réactifs utilisés.

6. DONNEES PHARMACEUTIQUES

6.1. Liste des excipients

Méglumine, eau pour préparations injectables.

6.2. Incompatibilités

En l'absence d'étude d'incompatibilité, ce médicament ne doit pas être mélangé avec d'autres médicaments.

6.3. Durée de conservation

3 ans.

6.4. Précautions particulières de conservation

Pas de précautions particulières de conservation.

6.5. Nature et contenu de l'emballage extérieur

Flacons de 5, 10, 15 et 20 mL en verre de type II, bouchés par un bouchon en élastomère.

Toutes les présentations peuvent ne pas être commercialisées.

6.6. Précautions particulières d’élimination et de manipulation

Préparer une seringue avec aiguille. Soulever le disque plastique. Perforer le caoutchouc du bouchon avec l'aiguille après avoir nettoyé le bouchon avec un tampon imbibé d'alcool.

Prélever la quantité de produit nécessaire à l'examen et l'injecter par voie intraveineuse.

7. TITULAIRE DE L’AUTORISATION DE MISE SUR LE MARCHE

GUERBET

BP 57400

95943 ROISSY CDG CEDEX

FRANCE

8. NUMERO(S) D’AUTORISATION DE MISE SUR LE MARCHE

 358 954-2: 5 mL en flacon (verre).

 331 713-4: 10 mL en flacon (verre).

 331 714-0: 15 mL en flacon (verre).

 331 715-7: 20 mL en flacon (verre).

9. DATE DE PREMIERE AUTORISATION/DE RENOUVELLEMENT DE L’AUTORISATION

[à compléter par le titulaire]

10. DATE DE MISE A JOUR DU TEXTE

[à compléter par le titulaire]

11. DOSIMETRIE

Sans objet.

12. INSTRUCTIONS POUR LA PREPARATION DES RADIOPHARMACEUTIQUES

Sans objet.

CONDITIONS DE PRESCRIPTION ET DE DELIVRANCE

Liste I.

Médicament soumis à prescription médicale.

## Annexe 3 lettre d’INFORMATION ET DE CONSENTEMENT

**LETTRE D’INFORMATION**

Lisez attentivement cette notice et posez toutes les questions qui vous sembleront utiles. Vous pourrez alors décider si vous voulez participer à cette recherche ou non.

Titre de l’étude : ETUDE MULTIPARAMETRIQUE DU REMODELAGE CARDIAQUE APRES INFRACTUS MYOCARDIQUE REVASCULARISE EN PHASE AIGUE : RELATION AVEC LES CONCENTRATIONS SERIQUES EN ALDOSTERONE

Nom du Promoteur : CHU de Nancy

Adresse du promoteur : 29, avenue du Maréchal de Lattre de Tassigny - 54035 NANCY cedex

Madame, Mademoiselle, Monsieur,

Vous avez été hospitalisé(e) dans le service de cardiologie suite à un **infarctus du myocarde**. La conséquence est qu’une partie plus ou moins importante de votre muscle cardiaque a été lésée et le principal risque est l’évolution vers **l’insuffisance cardiaque**. C’est ce qu’on appelle dans cette étude « le remodelage cardiaque » et il s’agit d’une dégradation progressive du fonctionnement cardiaque pouvant aboutir à une situation où le coeur ne peut pas toujours assurer un débit sanguin suffisant et vous pourriez en être gêné (fatigue, essoufflement …).

Le risque de cette évolution dépend avant tout de la sévérité de votre infarctus (la quantité de muscle cardiaque qui a été lésée) mais d’autres **paramètres peuvent** **accélérer l’évolution vers l’insuffisance cardiaque :** par exemple, un mauvais fonctionnement de vos vaisseaux ou de vos reins, et la sécrétion dans le sang de certaines hormones comme l’aldostérone. Il est particulièrement important de pouvoir étudier le rôle de l’aldostérone car nous disposons de traitements susceptibles de s’opposer à l’action de cette hormone. Cependant, de tels traitements ne pourront pas être réellement efficaces si on ne sait pas auparavant dans quelles conditions et chez quels types de patients l’aldostérone joue effectivement un rôle néfaste. Le but de cette étude est justement d’identifier ces conditions et ces types de patients.

L’étude à laquelle nous vous demandons de bien vouloir participer se propose **d’analyser la plupart des paramètres** jouant un rôle dans l’évolution vers l’insuffisance cardiaque grâce à : **1)** des prélèvements sanguins, qui permettront entre autre de mesurer la concentration d’aldostérone dans le sang, et **2)** la réalisation d’un examen d’IRM qui permettra rapidement (en moins d’une heure) de mesurer l’importance de votre infarctus et d’étudier très précisément le fonctionnement de votre coeur et de vos vaisseaux.

L’ensemble de ce bilan sera réalisé **dans les jours suivant votre infarctus puis 6 mois plus tard**, de manière à en suivre l’évolution.

Le docteur (Nom et prénom)…………….………………………………………………………

Adresse…………………………………………………………………………………………..

Téléphone…………………………

Praticien investigateur, vous propose de participer à l’étude intitulée «ETUDE MULTIPARAMETRIQUE DU REMODELAGE CARDIAQUE APRES INFRACTUS MYOCARDIQUE REVASCULARISE EN PHASE AIGUE : RELATION AVEC LES CONCENTRATIONS SERIQUES EN ALDOSTERONE ».

L’objectif essentiel de notre étude est de déterminer : 1) si la concentration d’aldostérone dans le sang est plus élevée chez les patients qui évoluent vers l’insuffisance cardiaque après un infarctus myocardique, 2) les mécanismes de survenue de cette action délétère de l’aldostérone et les circonstances de cette survenue (chez quels types de patients trouve-t-on cette action délétère ?).

Cette recherche se déroulera au sein du CHU de Nancy. Nous prévoyons d’y inclure 250 patients qui comme vous ont présenté un infarctus du myocarde datant de moins de 4 jours.

L’étude se déroulera de la façon suivante :

Après une lecture attentive de cette note d’information et si vous acceptez de participer, vous signerez le formulaire de consentement éclairé (en 3 exemplaires dont 1 vous sera remis).

Lors de votre inclusion, une évaluation clinique sera réalisée dans le service de cardiologie par le médecin investigateur de l’étude. Votre médecin recueillera également des informations relatives à votre santé : antécédents médicaux, pathologies actuelles et traitements en cours.

Un bilan initial sera alors organisé 2 à 4 jours après votre infarctus. Lors de ce premier bilan, les examens suivants seront réalisés :

- un prélèvement sanguin (prise de sang) d’environ 30 mL de sang, pour doser certaines molécules, qui peuvent elles aussi renseigner sur les mécanismes d’action de l’aldostérone sur l’aggravation d’un mauvais fonctionnement du cœur
- un examen IRM cardio-vasculaire (= imagerie par résonance magnétique). Le mot magnétique indique que l’appareil comporte un aimant, et c’est grâce à cet aimant que nous pourrons observer des images du cœur et des vaisseaux. Une injection intraveineuse d’un produit de contraste (Dotarem ^TM^) sera réalisée pour mesurer précisément l’importance de votre infarctus.

Vous devrez rester en station couchée pendant environ 60 minutes.

Le sentiment de malaise par crainte d’être enfermé (claustrophobie) est un problème courant bien connu du personnel des services IRM. Souvent on peut l’éviter par des moyens simples, sans aucun traitement. Si, par exemple, vous êtes mal à l’aise dans un ascenseur, parlez-en à votre médecin afin que le personnel du service IRM vous prenne particulièrement en charge.

- Puis un bilan à 6 mois (+/- 7 jours) sera ensuite organisé et les mêmes examens que ceux réalisés lors du bilan initial seront effectués, à savoir : le même prélèvement sanguin (prise de sang d’environ 30 mL de sang) et le même examen d’IRM avec l’injection du produit de contraste.

La durée totale de votre participation à cette étude sera donc de 6 mois après votre inclusion.

**Contraintes liées au protocole**

Les ponctions veineuses peuvent engendrer des désagréments.

Concernant le produit de contraste administré pour les IRM : des effets indésirables ont parfois été observés et ils sont le plus souvent mineurs (maux de tête, fourmillements, douleur au site d'injection, nausées, vomissements, …).

Des réactions allergiques sont aussi très rares mais possibles. Ces réactions sont le plus souvent limitées à des signes cutanés (démangeaisons, rougeur), mais des formes plus graves ont aussi été observées (asthme, oedème laryngé, état de choc …).

**Perspectives**

Cette étude doit nous permettre : 1) de mieux comprendre pourquoi certains patients évoluent défavorablement vers l’insuffisance cardiaque après un infarctus myocardique, 2) de préciser le rôle de l’hormone aldostérone dans cette évolution défavorable et 3) d’identifier les patients chez lesquels l’action de cette hormone pourrait être utilement combattue par des médicaments anti-aldostérone spécifiques.

Le Dr……………………… vérifiera que vous êtes bien affilié(e) à un régime de sécurité sociale.

Vous pourrez participer à une autre recherche durant votre participation à ce protocole.

Votre participation à cette étude est entièrement volontaire.

Votre refus de participer n’aura aucune conséquence sur le type et la qualité de votre prise en charge, ainsi que sur les relations avec le praticien investigateur. Si vous acceptez, vous pouvez à tout moment quitter cette étude sans conséquences sur votre futur traitement. Vous pouvez également sortir de l’étude si votre praticien pense que c’est mieux pour vous.

Toute nouvelle information devenant disponible au cours de l'étude et pouvant avoir des implications sur votre personne vous sera fournie par le Dr……………………

Le Promoteur prend en charge les frais supplémentaires liés à d’éventuels fournitures ou examens spécifiquement requis pour cette recherche.

Conformément à la réglementation en vigueur, un contrat d’assurance a été souscrit par le Promoteur.

A l’issue de la recherche, vous serez informé(e) des résultats globaux de la recherche par un courrier de l’investigateur coordonnateur, le Dr ANGIOI.

Le Comité Protection des Personnes Est III a émis un avis favorable initial à la réalisation de cette étude le XXXXXX. L’Agence Française de Sécurité Sanitaire des Produits de Santé (AFSSAPS) a donné son autorisation initiale pour la réalisation de cette étude le XXXXX.

En application de la loi « informatique, fichiers et liberté » du 06 janvier 1978 modifiée, le fichier informatique utilisé pour réaliser la présente recherche a fait l’objet d’une autorisation de la CNIL (Commission Nationale Informatique et Libertés). Seules les données nécessaires à la recherche seront recueillies. Vous avez néanmoins le droit de vous opposer à ce que les données vous concernant fassent l’objet d’un traitement automatisé. Si vous acceptez, vous aurez à tout moment le droit d’accéder aux données vous concernant. Vous aurez également le droit de demander à ce que les données inexactes ou devenues inexactes soient rectifiées. Vous pourrez à tout moment exercer ces droits auprès du Dr. ……………………….. (n° de téléphone)……………………………..

Pour toutes les informations de nature médicale, ces droits pourront être exercés directement ou par l’intermédiaire du médecin de votre choix.

Toutes les données et informations vous concernant resteront strictement confidentielles.

Les données vous concernant ne seront accessibles qu’aux personnes participant à cette recherche et aux personnes chargées par le promoteur de contrôler la qualité de l’étude ; le cas échéant elles pourront également être transmises aux autorités sanitaires habilitées. Dans tous les cas, elles seront exploitées dans les conditions garantissant leur confidentialité.

**FORMULAIRE DE CONSENTEMENT**

| Les détails concernant cette étude sont fournis dans la lettre d’information spécifique qui vous a été remise. Lisez attentivement cette notice et posez toutes les questions qui vous sembleront utiles. Si vous acceptez de participer à cette étude, veuillez compléter le formulaire ci-dessous. |
| --- |

Titre de l’étude : ETUDE MULTIPARAMETRIQUE DU REMODELAGE CARDIAQUE APRES INFRACTUS MYOCARDIQUE REVASCULARISE EN PHASE AIGUE : RELATION AVEC LES CONCENTRATIONS SERIQUES EN ALDOSTERONE

Nom du Promoteur : CHU de Nancy

Adresse du promoteur : 29, avenue du Maréchal de Lattre de Tassigny - 54035 NANCY cedex

Je soussigné (e), M……………………………………………………. (*nom complet en lettres capitales)* déclare avoir compris le but et les modalités de cette étude, qui m’ont été pleinement expliqués par le Docteur …………………………………………………..

J’ai reçu le formulaire d’information spécifique que j’ai eu la possibilité d’étudier avec attention.

Des réponses ont été apportées à toutes mes questions.

J’ai disposé d’un délai de réflexion avant de prendre ma décision.

Je pourrai participer à une autre recherche durant ma participation à ce protocole.

J’accepte de participer à cette recherche dans les conditions précisées dans le formulaire d’information ci-joint. Je demeure libre de quitter l’étude à tout moment sans que cela n’affecte la prise en charge médicale ultérieure. J’en informerai alors le Docteur ………………………..

Le fait de ne plus participer à cette recherche ne portera pas atteinte à mes relations avec mon praticien et ne remettra pas en cause la qualité des soins ultérieurs.

J’ai été informé(e) que conformément à la réglementation sur les études cliniques, le Comité de Protection des Personnes Est III a rendu un avis favorable initial pour la réalisation de cette étude en date du XXXXX et l’Agence Française de Sécurité Sanitaire des Produits de Santé (AFSSAPS) a donné son autorisation initiale pour la réalisation de cette étude en date du XXXXXX.

J’ai également été informé que conformément à la loi en vigueur, un contrat d’assurance a été souscrit par le promoteur de la recherche.

Toutes les données me concernant, y compris mon dossier médical, resteront confidentielles. Je n’autorise leur consultation que par les personnes qui collaborent à la recherche, aux personnes chargées par le promoteur de contrôler la qualité de l’étude ainsi que par un représentant des autorités de santé.

J’accepte que les données nécessaires à la recherche soient recueillies durant ma participation à l’étude et fassent l’objet d’un traitement informatisé autorisé par la Commission Nationale Informatique et Liberté. J’ai bien été informé de la finalité du traitement (on m’a expliqué à quoi serviraient ces données) ainsi que des destinataires de ces données.

J’ai bien noté qu’en application de la loi « Informatique et Libertés » du 6 janvier 1978 modifiée, je dispose d’un droit d’accès aux données me concernant ainsi qu’un droit de rectification. Je peux exercer ces droits à tout moment auprès du Dr. ANGIOI – CHU de Nancy – Service de Cardiologie

Hôpital de Brabois - Téléphone : 03 83 15 32 40

Je donne mon consentement pour participer à cette recherche.

Je pourrai à tout moment demander toute information complémentaire au Dr. ………………. N° de téléphone …………………………

Mon consentement ne décharge en rien l’investigateur et le promoteur de l’ensemble de leurs responsabilités et je conserve tous mes droits garantis par la loi.

A l’issue de la recherche, je serai informé(e) des résultats globaux de cette recherche par un courrier de l’investigateur coordonnateur.

| A REMPLIR PAR LE PATIENT |
| --- |
| Nom : ……………….  Date : ………………..  Signature du patient |

| A REMPLIR PAR L’INVESTIGATEUR | |
| --- | --- |
| Je soussigné Docteur ………………………. (nom en lettres capitales) confirme avoir pleinement expliqué au patient et à son représentant légal le cas échéant le but et les modalités de cette étude ainsi que ses risques potentiels. Je m’engage à faire respecter les termes de ce formulaire de consentement, conciliant le respect des droits et des libertés individuelles et les exigences d’un travail scientifique.  N° de téléphone de l’investigateur : …………………………………….. | |
| Signature de l’investigateur : | Date : …………………………….. |

Fait en trois exemplaires dont un sera conservé par l’investigateur, un autre remis au patient et un conservé par le promoteur.

## Annexe 4 Fiche d’eig

Réservé Promoteur :ID

| **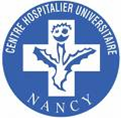** | **Cellule de promotion CHU de NANCY** | **DECLARATION D’EVENEMENT INDESIRABLE GRAVE**  **Cette fiche est à faxer dûment complétée dans les 24 heures ouvrées au 03 83 85 98 14** |
| --- | --- | --- |

| Identification du protocole :  Référence de l’étude :  **Titre :**    **Promoteur : CHU de NANCY**  Centre n/ Site : |_|_|, adresse :  téléphone : 03 83 15 39 09 | 1^ère^ notification  date de 1^ère^ notification :  _ _ / _ _ / _ _ _ _  Rapport de suivi n° : |_|  date de rapport de suivi :  _ _ / _ _ / _ _ _ _ |
| --- | --- |

| **Patient**  Nom : |_|_|_|  Prénom : |_|_|_|  N° Inclusion |_|_|  Sexe :  F  M  Date de naissance : _ _ / _ _ / _ _ _ _  Âge (ans) : Poids (kg) : |_|_|_| Taille (cm) : |_|_|_|  Date d’entrée dans l’étude _ _ /_ _/_ _ _ _  Bras* Etude en aveugle oui non  *si applicable | **L’événement indésirable grave a entraîné** :  décès  mise en jeu du pronostic vital  nécessité d’hospitalisation ou de prolongation d’hospitalisation  invalidité/ incapacité temporaire ou permanente  anomalie, malformation congénitale  autres toxicités médicalement significatives (préciser) |
| --- | --- |

Médicament(s) étudié(s) suspecté(s)

| Nom commercial ou DCI | Forme | Dosage | Voie | Posologie | Date de 1ère administration | Date d’administration avant l’EIG |
| --- | --- | --- | --- | --- | --- | --- |
|  |  |  |  |  | _ _ /_ _ /_ _ _ _ | _ _ /_ _ /_ _ _ _ |
|  |  |  |  |  | _ _ /_ _ /_ _ _ _ | _ _ /_ _ /_ _ _ _ |

Médicaments associés/concomitants (à l’exclusion de ceux utilisés pour traiter l’événement)

| Nom commercial ou DCI | Forme | Dosage | Voie | Posologie | Date de début | Date de fin | indication |
| --- | --- | --- | --- | --- | --- | --- | --- |
|  |  |  |  |  | _ _ /_ _ /_ _ _ _ | _ _ /_ _ /_ _ _ _ |  |
|  |  |  |  |  | _ _ /_ _ /_ _ _ _ | _ _ /_ _ /_ _ _ _ |  |
|  |  |  |  |  | _ _ /_ _ /_ _ _ _ | _ _ /_ _ /_ _ _ _ |  |
|  |  |  |  |  | _ _ /_ _ /_ _ _ _ | _ _ /_ _ /_ _ _ _ |  |
|  |  |  |  |  | _ _ /_ _ /_ _ _ _ | _ _ /_ _ /_ _ _ _ |  |
|  |  |  |  |  | _ _ /_ _ /_ _ _ _ | _ _ /_ _ /_ _ _ _ |  |

Antécédents/maladies concomitantes (autres que l’indication du médicament étudié) :__

**Informations complémentaires :**

**Antécédents médicaux/chirurgicaux/thérapeutiques notoires du patient** (éléments pouvant avoir un retentissement sur l’évènement indésirable ou le traitement ex allergie, insuffisance rénale…)

Réservé Promoteur :ID

**Résultats des examens complémentaires** (indiquer les tests et les résultats, joindre les photocopies)

**Déclaration d’évènement indésirable grave (recto)**

**Etude :**

**Initiales patient :** Nom : |_|_|_| Prénom : |_|_|_| N° Inclusion |_|_|

**Désignation de l’évènement indésirable grave**: ________________________________________________________________________________________________

Date de début : _ _ / _ _ / _ _ _ _ et heure de survenue : |_|_| H |_|_| min

Délai de survenue après l’initiation du traitement : ( jours  mois  années)

Description :(Diagnostic ou symptômes prédominants. Décrire la chronologie de l’événement et les mesures thérapeutiques qui ont été prises. Joindre les comptes-rendus anonymisés d'hospitalisation d'examens et/ou résultats de laboratoire si nécessaire)

__________________________________________________________________________________________________________

__________________________________________________________________________________________________________

__________________________________________________________________________________________________________

__________________________________________________________________________________________________________

__________________________________________________________________________________________________________

__________________________________________________________________________________________________________

__________________________________________________________________________________________________________

__________________________________________________________________________________________________________

| Action prise | L’évènement s’est-il amendé après arrêt ou modification du traitement ? | L’évènement a-t-il réapparu après réintroduction du traitement ? | Causalité |
| --- | --- | --- | --- |
| Aucune  Diminution de la posologie  Augmentation de la posologie  Interruption du traitement  Arrêt du traitement  Recours à une action thérapeutique | Oui  Non  Recul insuffisant  Non applicable | Oui  Non  Non applicable | Evènement lié au traitement  Evènement non lié au traitement  Ne peut conclure |

| **Évolution**  sujet non encore rétabli  guérison sans séquelle Date de la guérison : _ _ / _ _ / _ _ _ _  guérison avec séquelles  inconnue | **En cas de décès** : date du décès : _ _ / _ _ / _ _ _ _  décès sans rapport avec l’effet  décès auquel l’effet a pu contribuer  décès dû à l’effet |
| --- | --- |

Nom de l’investigateur Date _ _/_ _/_ _ _ _ Signature

Cadre réservé au promoteur

EIG n° :________________

Date de réception par le promoteur : _ _ / _ _ / _ _ _ _

Levée de l’aveugle :  oui  non  sans objet Résultat de la levée de l’aveugle : ________________________

***Selon le promoteur, l’événement est plutôt lié :***  au(x) traitement(s) à l’essai  à la progression de la maladie

au protocole de l’essai  au(x) autre(s) maladie(s) concomitante(s)

autre(s) traitement(s) concomitant(s)  autre(s) :……………………………...

***Si selon le promoteur, l’événement semble plutôt lié au(x) médicament(s) étudié(s) :***

L’effet indésirable est attendu inattendu

Commentaires du promoteur
