# Supplementary material for: Plasma Galectin-3 predicts deleterious vascular dysfunction affecting post-myocardial infarction patients: An explanatory study
Source: PLoS One. 2020 May 11;15(5):e0232572. doi: 10.1371/journal.pone.0232572 (PMC7213735; doi:10.1371/journal.pone.0232572)
Supplement: S2 Protocol — (DOCX) [file pone.0232572.s004.docx]

**Study protocol based on that registered on the ClinicalTrials.gov site** (NCT01109225) **and extracted from the French document approved by the local Ethics Committee** (agreement n° 2009-A00537-50 from the “comité de protection des personnes se prêtant à la recherche biomédicale (CPP))

Brief Title **relation between aldosterone and cardiac REmodeling after Myocardial Infarction (REMI cohort)**

Official Title: **Multiparametric Study of Cardiac Remodeling After Myocardial Infarction Revascularized in Acute Phase: Relation With the Serum Concentrations in Aldosterone**

First Submitted Date: April 22, 2010

Brief Summary: This study aims to determine whether aldosterone blood levels are predictive of cardiac remodeling at 6 months following myocardial infarction with ST elevation (STEMI), independently of conventional predictive factors (size of myocardial infarction, age, hypertension, etc.) in revascularized patients during the acute phase of MI.

Detailed Description: After myocardial infarction, the evolution toward cardiac failure is generally linked to a progressive worsening of cardiac dysfunction and remodeling. Cardiac remodeling is largely the consequence of myocardial injury due to myocardial infarction, although other parameters including age, arterial hypertension, etc. may also represent important predictors. Aldosterone blood levels measured within the first hours of admission for myocardial infarction is associated with increased rates of adverse outcomes. Aldosterone acts on wound healing and fibrosis processes in the myocardium as well as on systemic volemia.

Original Primary Outcome Measures: aldosterone blood concentration

Current Primary Outcome Measures: Cardiac remodeling within 6 months of myocardial infarction as defined as the change in left ventricle volumes (increase of more than 20 % of the diastolic volume of the left ventricle) [Time Frame: from initial assessment to 6 months]

Current Secondary Outcome Measures:

- Vascular and cardiac parameters measured during the initial assesment and at 6 months (both functional and structural - by echocardiography or MRI) [Time Frame: from initial assessment to 6 months ]
- Long-term cardiac remodeling measured by echocardiography at 3 to 9 years of initial enrollment [Time Frame: at 3 to 9 years of initial enrollment]

Original Secondary Outcome Measures: cardiac MRI [ Time Frame: april 2012 ]

Study Type Interventional

Study Design Intervention Model: Single Group Assignment

Masking: None (Open Label)

Primary Purpose: Diagnostic

Condition Myocardial Infarction

Experimental: Myocardial infarction

All patients. Patients hospitalized for a first myocardial infarction with known shift of the segment ST revascularized in acute phase by primary angioplasty and dated less than 4 days.

Original Estimated Enrollment: (submitted: April 22, 2010) 250

Inclusion Criteria:

- Man or woman hospitalized for a first myocardial infarction with known shift of the segment ST revascularized in acute phase by primary angioplasty and dated less than 4 days
- Patient presenting a stable clinical state
- Patient presenting a regular sinusal cardiac rhythm
- Patient having an age ≥ 18 years
- Main exclusion criteria were: The patients were referred to a medical check-up involving MRI at 2 to 4 days after acute MI reperfusion and 6 months (± 15 days) later.

Exclusion Criteria:

- Previous history of MI,
- Any other significant cardiac disease (including > grade-2 mitral regurgitation),
- Any contraindication to cardiac MRI,
- Absence of sinus cardiac rhythm,
- Multivessel disease at coronary angiography
- A >12h delay-time between the onset of chest pain and reperfusion
- Counter-indication with examination MRI
- Severe claustrophobia
- Antecedent of over-sensitiveness to gadolinium salts
- Nonischaemic Cardiopathy
- Cardiac surgery planed in the 6 months
- Women into old to procreate without effective contraception

Sexes Eligible for Study: All

Ages: 18 Years and older (Adult, Older Adult)

Accepts Healthy Volunteers: No

Intervention: The patients were referred to a medical check-up involving MRI at 2 to 4 days after acute MI reperfusion and 6 months (± 15 days) later.

- Biological: plasma biomarkers of heart failure (Brain Natriuretic Peptide (BNP)), of myocardial necrosis (peak Creatine Kinase-MB and peak Troponin) and of systemic inflammation and/or RASS activation (C-Reactive Protein, Neutrophil Gelatinase-Associated Lipocalin (NGAL), Galectin-3, active Renin and Aldosterone.
- Procedure: MRI exams performed on a single 3.0 Tesla magnet (Signal HDxt, GE Healthcare, Milwaukee, Wisconsin) with a cardiac coil. Systolic, diastolic and mean brachial blood BP are measured with an automated sphygmomanometer.
- A steady-state free precession pulse sequence is used to assess cardiac function in contiguous short axis planes, and LV end-diastolic volume, end-systolic volume, LV mass and ejection fraction are obtained using dedicated software.
- The MI area is analyzed on short axis slices covering the LV volume and recorded with a T1-weighted segmented phase-sensitive inversion-recovery (PSIR) sequence, 10 to 15 minutes after the injection of a gadolinium-labeled tracer (0.1 mmol.kg-1 of body weight of Dotarem®, GUERBET, France).
- Aortic stroke volume (SV) indexed to body surface area is determined in the ascending aorta by using a velocity-encoded phase-contrast gradient-echo sequence and the “CV flow” quantification and is used to calculate cardiac index (SV x heart rate) and systemic vascular resistance (SVR: mean BP / cardiac index).
- Procedure: echocardiography
- Biological: urine sample
- Procedure: pulmonary echography
- Procedure: vascular check
- Procedure: renal echography

Listed Location Countries: France

Responsible Party: Central Hospital, Nancy, France

Study Sponsor: Central Hospital, Nancy, France

Principal Investigator: Nicolas GIRERD, Doctor Centre d'Investigation Clinique Plurithématique 1433/INSERM/CHRU de Nancy

PRS Account : Central Hospital, Nancy, France

Publications

1. Huttin O, Mandry D, Eschalier R, Zhang L, Micard E, Odille F, Beaumont M, Fay R, Felblinger J, Camenzind E, Zannad F, Girerd N, Marie PY. Cardiac remodeling following reperfused acute myocardial infarction is linked to the concomitant evolution of vascular function as assessed by cardiovascular magnetic resonance. J Cardiovasc Magn Reson. 2017 Jan 4;19(1):2. doi: 10.1186/s12968-016-0314-6.
2. Huttin O, Marie PY, Benichou M, Bozec E, Lemoine S, Mandry D, Juillière Y, Sadoul N, Micard E, Duarte K, Beaumont M, Rossignol P, Girerd N, Selton-Suty C. Temporal deformation pattern in acute and late phases of ST-elevation myocardial infarction: incremental value of longitudinal post-systolic strain to assess myocardial viability. Clin Res Cardiol. 2016 Oct;105(10):815-26. doi: 10.1007/s00392-016-0989-6. Epub 2016 Apr 23.
3. Huttin O, Petit MA, Bozec E, Eschalier R, Juillière Y, Moulin F, Lemoine S, Selton-Suty C, Sadoul N, Mandry D, Beaumont M, Felblinger J, Girerd N, Marie PY. Assessment of Left Ventricular Ejection Fraction Calculation on Long-axis Views From Cardiac Magnetic Resonance Imaging in Patients With Acute Myocardial Infarction. Medicine (Baltimore). 2015 Oct;94(43):e1856. doi: 10.1097/MD.0000000000001856.
4. Lemarié J, Huttin O, Girerd N, Mandry D, Juillière Y, Moulin F, Lemoine S, Beaumont M, Marie PY, Selton-Suty C. Usefulness of Speckle-Tracking Imaging for Right Ventricular Assessment after Acute Myocardial Infarction: A Magnetic Resonance Imaging/Echocardiographic Comparison within the Relation between Aldosterone and Cardiac Remodeling after Myocardial Infarction Study. J Am Soc Echocardiogr. 2015 Jul;28(7):818-27.e4. doi: 10.1016/j.echo.2015.02.019. Epub 2015 Mar 31.
5. Huttin O, Lemarié J, Di Meglio M, Girerd N, Mandry D, Moulin F, Lemoine S, Juillière Y, Felblinger J, Marie PY, Selton-Suty C. Assessment of right ventricular functional recovery after acute myocardial infarction by 2D speckle-tracking echocardiography. Int J Cardiovasc Imaging. 2015 Mar;31(3):537-45. doi: 10.1007/s10554-014-0585-7. Epub 2015 Jan 6.
